# Targeted phytotherapy: essential oils, multi-target cancer action & nanodelivery

**DOI:** 10.3389/fonc.2026.1913688

**Published:** 2026-07-29

**Authors:** Kali Prasad Pattanaik, Laxmipriti Behera, Usha Rani Panda, Satyabrata Moharana, Prajakta Tambe, Jagannath Prasad Tripathy, Sanatan Majhi, Rout George Kerry, Sanghamitra Nayak

**Affiliations:** 1 R&D Department, BioPioneer Pvt. Ltd., KIIT-TBI, KIIT DU, Patia, Bhubaneswar, India; 2 Department of Bioinformatics, Orissa University of Agricultural Technology, Bhubaneswar, India; 3 Department of Biotechnology, College of Pharmaceutical Sciences, Berhampur, Odisha, India; 4 Department of Biotechnology, College of Pharmaceutical Sciences, Mahuda, Berhampur, Odisha, India; 5 Advanced (magnetic) Theranostic Nanostructures Lab, International Iberian Nanotechnology Laboratory, Braga, Portugal; 6 Department of Biotechnology, Fakir Mohan University, Vyasa Vihar, Nuapadhi, Balasore, Odisha, India; 7 Department of Biotechnology, Utkal University, Bhubaneswar, Odisha, India; 8 Centre for Biotechnology, Siksha ‘O’ Anusandhan (Deemed to be University), Bhubaneswar, Odisha, India

**Keywords:** anticancer mechanisms, essential oils, multi-targeted therapy, nanoformulation, precision phytotherapy

## Abstract

Biochemical components of essential oils (EOs) are an innovative but largely underexplored type of precision phytotherapy (PT) in oncology owing to their anticancer activity against many types of tumors. This article discusses the important multitargeted molecular mechanisms of EOs (apoptosis, cell cycle arrest, angiogenesis, metastasis, tumor microenvironment) and their role as antitumor agents. In particular, an effort has been made to emphasize on the most notable volatile EOs such as thymol, eugenol, limonene, β-caryophyllene, and their interaction with oncogenic signaling pathways (NF-κB, PI3K/AKT/mTOR, MAPK, STAT3). Additionally, the ability of EO constituents to act in synergy with standard chemotherapeutic regimens and immune checkpoint inhibitors are also emphasized. In order to help provide evidence for the anticancer activity of EOs by identifying and examining new nanotechnology-based strategies for drug delivery that address the physicochemical challenges of EOs, this article evaluates advanced nanoformulation techniques, such as lipid-based nanoparticles, polymeric micelles, nanoemulsions, and mesoporous silica carriers. This is discussed in order to define how passive (diffusion) or active (specific targeting) methods can be utilized to improve delivery/absorption. Moreover, this article also discusses EO-derived phytocompounds that can be used in combination with existing chemotherapy or immunotherapy to provide a mechanism(s) to ultimately reverse multidrug resistance. Finally, recent translational bottlenecks before integrating EOs-based PT into precision oncology, such as standardization, regulatory framework, and clinical trials are also addressed.

## Graphical Abstract

**Figure:**
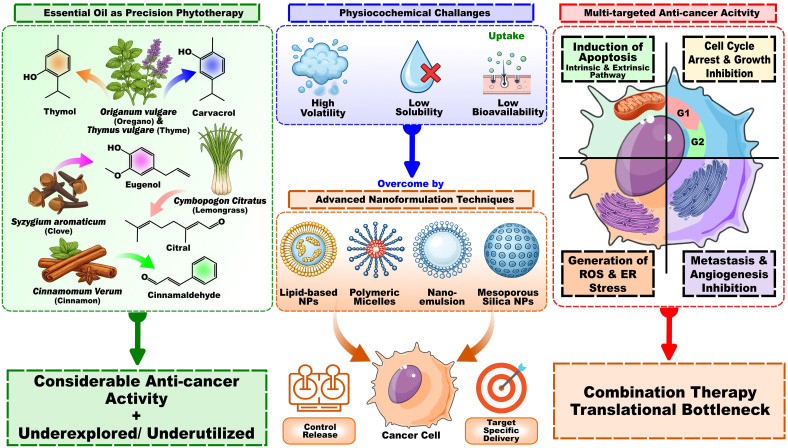

## 1 Introduction

For centuries, essential oils (EOs) have been used as a part of traditional healing systems, such as Ayurveda and Traditional Chinese Medicine for their fungal and bacterial fighting, reducing inflammation, and providing pain relief (1). Historically, EOs were either inhaled, applied externally to the skin, or ingested for healing wounds (2), treating stomach problems or controlling pain (3). Recent research has also focused on volatile plant extracts and their unique makeup as a source of bioactive molecules that have been found to have anti-proliferative effects in preclinical studies (4). The shift from traditional to evidence-based oncology for EO is driven by advanced tools like chromatography-mass spectrometry (GC-MS). This technique provides a precise profile of the compounds in an EO, and high-throughput screening tests that identify which EOs selectively kill malignant cells (5). For example, EOs extracted from *Origanum vulgare* (6) and *Melaleuca alternifolia* (7) has shown to suppress growth of cancer cells in a dose-dependent manner while also reducing oxidative stress and inflammatory response.

The discovery that EOs can be used as lead compounds for precision phytotherapy has led to a paradigm shift towards developing drugs from natural sources. Recent preclinical studies have shown that EOs induce selective apoptosis in cancer cells (8), while sparing normal epithelial cells, due to the different composition of lipids in the membranes of cancerous cells (9). Further, the increased vulnerability of these transformed cells to metabolic stress is evident from their elevated basal levels of reactive oxygen species. Therefore, as oncology increasingly embraces the use of low-toxicity, multi-agent therapies that can address redundancy and resistance in signalling pathways (10), there is an excellent opportunity for EOs to bridge traditional knowledge with current practices in molecular oncology (11). The volatile characteristics of EOs will require the development of novel delivery systems for optimal therapeutic effects without compromising safety or stability/efficacy (12). Understanding EOs’ historical medicinal use and modern validation via chemical/biological methods informs their integration into contemporary cancer protocols. This continuum of traditional and scientific knowledge provides evidence of both the ongoing value of plant-based remedies as well as the necessity for modern evidence-based research that will translate traditional knowledge regarding plant-based remedies into scientifically validated therapeutic solutions for patient care.

Currently, the research community is witnessing evolutionarily similar systems across traditional botanical medicines (phytotherapy) that is evolving physiological phenomenon (13), molecular oncology, and nanomedicine (14). Here, the basis for delivering plant-based bioactives is they that will have better specificity for targets, predictability of pharmacokinetics, and stratification of patients based on molecular profiles of tumors than conventional chemotherapy. Precision phytotherapy can achieve these goals by using the polypharmacological aspects (15) of the phytochemical spectrum to affect multiple oncogenic pathways at once and simultaneously reduce systemic toxicities (16). Additionally, EO represent paradigms of this system because they contain complex mixtures of terpenes and phenylpropanoids (17) that can achieve multiple biological networks due to combination of several components within one compound. The precision aspect is considered when each EO constituent is fully characterized chemically using methods such as gas chromatography/mass spectrometry, nuclear magnetic resonance, and mechanistically mapped using proteomic and transcriptomic analyses. The compounds can then be delivered using engineered nanocarriers (18) designed to achieve (1) targeted accumulation (19) (2), controlled release kinetics (20), and (3) optimized dosing regimens individually tailored to each patient’s needs (21, 22). This comprehensive strategy addresses the traditional limitations associated with utilizing crude botanical extracts including significant lot-to-lot variability and unpredictable bioavailability due to metabolic variations.

**Figure 1:**
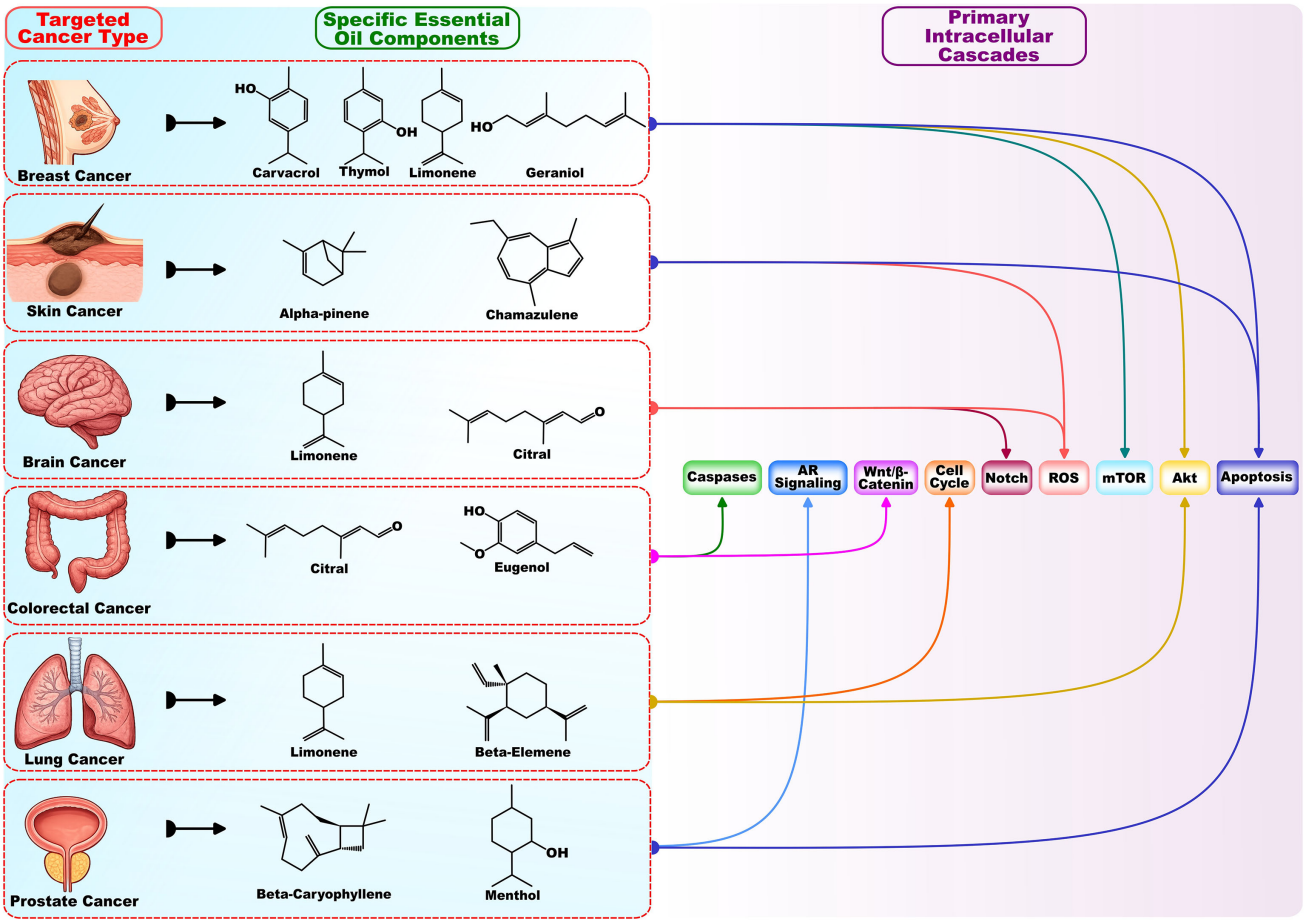
Targeted cancer types, the EO components associated with them, and the primary intracellular cascades are summarized in the figure. The relationships between the cancer types (breast cancer and brain cancer), the essential oil derived bioactive components that relate to each of these cancer types, and the major cellular pathways or molecular cascades associated with those bioactive components are summarized in this figure. For breast cancer, the components include carvacrol, thymol, limonene, and geraniol, and the associated cascades are caspases, AR signalling, Wnt/β-catenin, cell cycle, Notch, ROS, mTOR, Akt, and apoptosis. The figure includes repeated entries for those components (limonene, citral, eugenol) as they were presented in the original figure. For brain cancer, the same components and cascades are included; however, some of the entries are abbreviated or repeated (i.e. limone, citrat). The relationship between the signalling of essential oils and different forms of cancer is complex and overlapping as indicated in the figure. The components of the figures were assembled in Inkscape 1.3.2 (091e20e, 2023-11-25, custom).

Pharmacogenomic insights have been incorporated into personalized phytotherapy (23) and integrative medicine, indicating that variations in cytochrome P450 enzyme activity and drug transporter expression can greatly affect the effectiveness, metabolism, and safety of EO constituents (24, 25). Matching EO-derived compounds to established molecular subtypes of cancer, involves correlating different phytocompound profiles with established driver mutations, such as EGFR overexpression or PI3K pathway hyperactivation (**Figure 1**). Theoretically, this systematic correlation would enable clinicians to match phytocompounds to oncogenic driver mutations. Rational combinations of EO bioactives with existing targeted therapies or immune checkpoint inhibitors (26), in order to exploit synergistic vulnerabilities in both cancer cells and to their microenvironment, are also highly emphasized in personalized phytotherapy (27, 28). The discipline is evolving rapidly from being predominantly empirical herbalism to being a robust evidence-based disciplinary area, relying on advanced omics technologies. Moreover, computational molecular docking simulations, and complex pharmacokinetic/pharmacodynamic models have been incorporated to optimize the design of EO formulations (29). The regulatory frameworks are continuing to evolve quickly based on the distinctive properties of various complex natural products. Personalized phytotherapy is a means of significantly broadening the arsenal of therapies for oncology, in a manner that is affordable and scalable. The conceptual framework of personalized phytotherapy establishes the use of plant-derived medicines as more than just alternative or adjunctive therapies. They have the potential to be complementary therapies that increase the total therapeutic index of medicines, delay or overcome mechanisms of acquired resistance, and enhance the quality of life of patients with cancer undergoing complex treatment regimens.

## 2 Chemical composition and bioactive constituents of anticancer essential oils

The sources of EOs are primarily from so-called “terpenoids” and “phenylpropanoids” (17), but since anywhere from 100 to 500 individual compounds (30) have been identified in EOs from plant sources, “they” will contain both very different types of EO-based compounds. The therapeutic benefits from EOs will depend on their highly specific molecular (cellular) and chemical fingerprint. The major fractions of EOs that have anticancer activity are: monoterpenes, sesquiterpenes, and aromatic aldehydes (31, 32), and each of those three classes of EOs will have very different profiles of pharmacodynamic effects. Thus, it is crucial to conduct standardized phytochemical characterization of EOs using multiple orthogonal analytic techniques in both laboratory and clinical investigations of EOs.

### 2.1 Terpenoids: monoterpenes and sesquiterpenes

Anticancer EOs are terpenoids that are further divided into categories called monoterpenes (C10) (33) and sesquiterpenes (C15) (34) according to their total isoprene units. The low molecular weight monoterpenes such as limonene, α-pinene, and linalool facilitate rapid cellular absorption (35, 36). Intracellularly, these compounds are known to disrupt mitochondrial membrane potential (37, 38) subsequently activating downstream caspases to induce apoptosis (39). For instance, limonene, abundant in citrus peel oils, exhibits robust anticancer effects across various preclinical breast models (40) [e.g., breast (41) and liver cancer models (42)]. Its anti-proliferative mechanisms include upregulation of p21 (43), inhibition of RAS-protein prenylation (44), and suppression of the PI3K/AKT signal transduction pathway (45). Sesquiterpenes, on the other hand, have larger molecular weights than monoterpenes, giving them a greater degree of specificity for target sites in the body, including nuclear receptors and key inflammatory enzymes (46). β-caryophyllene, one of the sesquiterpenes that can activate the CB2 receptor, may reduce chronic inflammation related to tumors (47) and increase the likelihood of reducing cell proliferation related to tumors (48). A few reports suggest that structural modification can alter the functions of the components, e.g., incorporation of different types of functional groups (hydroxyls, carbonyls, and epoxides) create differences in redox potential (49), protein-receptor binding capacity (50), and also the potential biological activities associated with the components (51). Studies using chiral chromatography, for example, also suggest enantiomer specific anticancer effects emphasizing the importance to resolve stereochemistry of each component during production of phytotherapeutics and adequate control of their quality postproduction. The continued development of terpenoid-rich EOs as potential cancer treatment options by mapping the full oncogenic proteomes is essential to the scientific basis needed to promote their use as chemotherapeutic agents in precision oncology regimens.

### 2.2 Phenylpropanoids and other volatile compounds

Phenylpropanoids are a large class of plant metabolites unified by a common C_6_–C_3_ framework, which pairs a six-carbon aromatic ring with a three-carbon side chain (52). Eugenol (53), chavicol (54), and safrole (55) are three examples of phenylpropanoids that may possess anticancer properties. Eugenol is found most abundantly in the EOs of clove and basil and has been shown to be very cytotoxic by causing fragmentation of DNA (53), inhibiting COX-2 (56), and modifying various inflammatory pathways (53, 56). Some studies have also suggested that eugenol may exert an indirect epigenetic effect through histone acetylation (57), especially when combined with other agents. In terms of its potential application in oncology research, however, eugenol is most valued as a pro-oxidant/antioxidant (58) and an anti-inflammatory agent (59), both of which contribute to a selective increase in stress levels within tumor cells. Similarly, hydroxychavicol a derivative of chavicol has also shown to induce apoptosis in CML cells selectively through mitochondrial depolarization through a ROS-mediated mechanism (60). Different studies have chemically-altered safrole and its derived compounds to decrease the potential for genotoxicity (61) while still preserving their anti-proliferative effects (62). The addition of other volatile compounds, such as some aldehydes and alcohols, to EOs also adds to the overall poly-pharmacological profile of EOs by means of providing additional/synergistic effects on disrupting cellular membranes (63), causing redox imbalances and interfering with cell signaling. As such, comprehensive profiling of these phenylpropanoid compounds is critical, as the abundance of each phenylpropanoid compound correlates directly with the total biological activity and the safety margin of any EO chemotype being investigated for anticancer purposes.

### 2.3 Structure–activity relationships

Studies on the structure-activity relationships of the constituents found within EOs indicate that three factors play a critical role in establishing the anticancer effectiveness of an oil constituent, as well as its target specificity. These three factors are the molecular topology (64), the strategic positioning of functional groups (65), and the overall lipophilicity (66) of an oil constituent. For instance, when referencing hydroxylated monoterpenes, known to contain an increased capacity to form hydrogen bonds (67) could provide the opportunity for effective interaction with the ATP-binding pocket of several kinases. In contrast, epoxidized terpenoids contain an increased amount of electrophilicity (68) and possess the ability to covalently modify thiol groups (69) on key cysteine residues of redox-sensitive proteins, such as Keap1. This modification of Keap1 can trigger activation of the Nrf2 signalling pathway, resulting in significant chemopreventive potential in normal cells. However, caution must be exercised when interpreting the effects of Nrf2 activation in pre-existing tumors due to the “Nrf2 paradox” and the potential for activated Nrf2 signalling pathway to favourably promote the survival and adaptation of cancer cells in the presence of oxidative stress (70, 71). Additional structure-activity relationship data demonstrate that stereochemistry contributes to the anticancer properties of terpenoids, whereby enantiomers of the same terpenoid can have differing activities and selectivity with respect to cancer cells (72, 73). In general, structural information can provide relevant information and rationale for the semi-synthetic creation of novel oil constituent candidates and for the design of novel nanoformulations (74) that enhance or preserve intended pharmacodynamic activities while providing improved delivery characteristics.

## 3 Molecular targets and anticancer mechanisms

**Figure 2:**
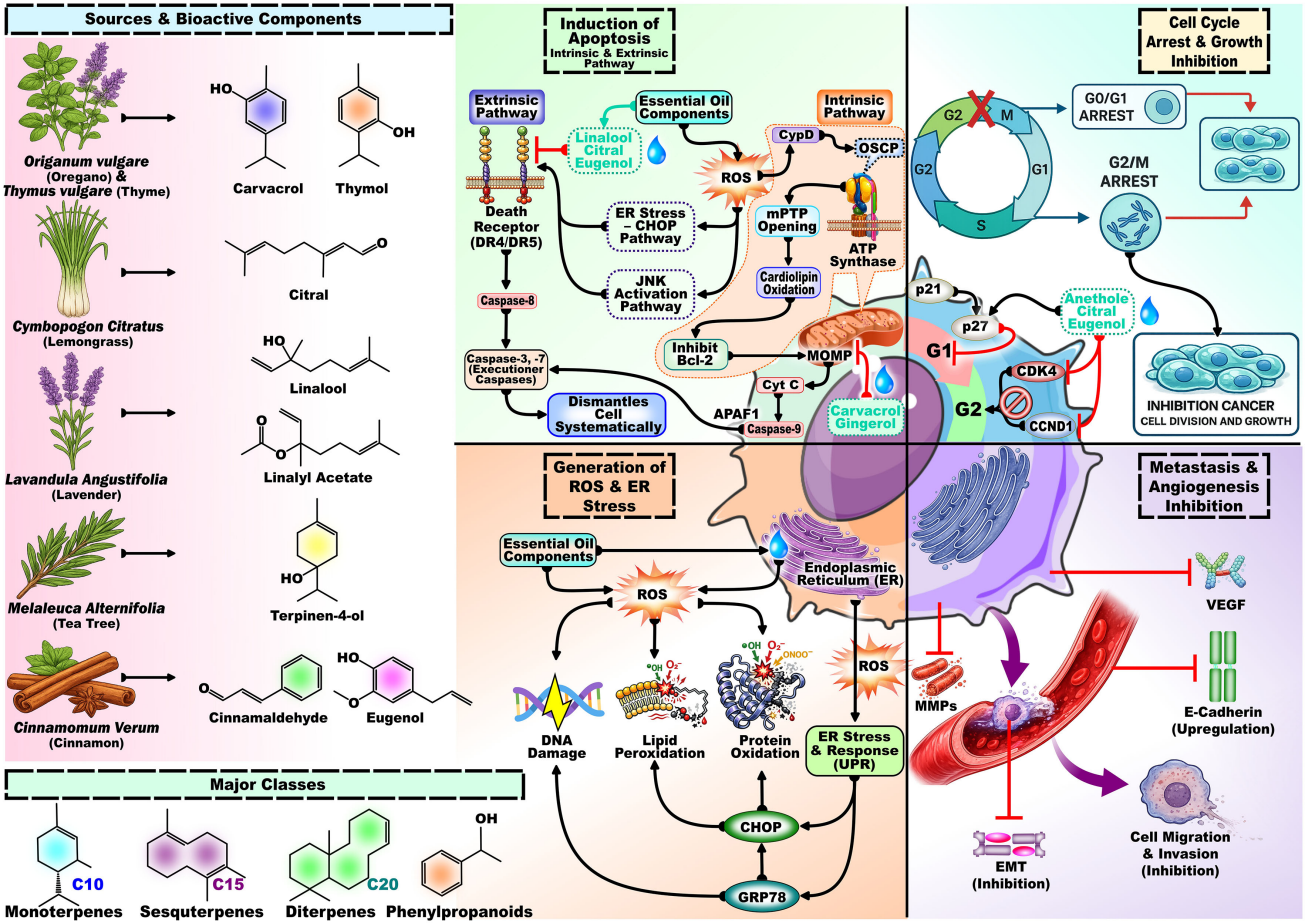
Illustration of possible mechanisms by which cancer cell proliferation can be inhibited by essential oils and their individual components. Some of the most frequently used essential oils as cancer treatments include: Origanum vulgare, Thymus vulgaris, Cymbopogon citratus, Lavandula angustifolia, Melaleuca alternifolia, and Cinnamomum verum, along with several of the essential oil active components: carvacrol, thymol, citral, linalool, linalyl acetate, terpinen-4-ol, cinnamaldehyde, and eugenol. The anticancer properties of these phytochemicals are mainly produced by oxidative and endoplasmic reticulum stress arising from reactive oxygen species (ROS), activation of both intrinsic and extrinsic pathways of apoptosis, cell cycle arrest, and inhibition of tumor growth, as well as inhibiting metastasis and angiogenesis through modulation of many different cellular pathways, including epithelial - mesenchymal transition (EMT), matrix metalloproteinases (MMPs), VEGF signaling pathways, and cell migration; and thus, restricting cancer metastasis and angiogenesis and delaying the advancement of cancer through the actions of these phytochemicals on such specific cellular pathways. The components of the figures were assembled in Inkscape 1.3.2 (091e20e, 2023-11-25, custom).

EOs exhibit anticancer activity by affecting many important cellular processes in multiple ways. The lipid-soluble nature of EOs permits access into cells allowing for modulation of protein expression levels (75), disruption of cellular organelle function (76), and alteration of various signalling pathways (8). Instead of working on a single target or pathway, EO constituents usually modulate multiple overlapping molecular networks that lead to programmed cell death (apoptosis), halting cellular proliferation, and causing significant changes in the structure of the tumor microenvironment (32). This polypharmacological characteristics of EO compounds reduce the compensatory resistance mechanisms that are frequently observed with traditional single target treatment approaches. An understanding of these multilayered mechanistic actions will be necessary for rational design and incorporation of EO-based treatments into broader precision oncology therapeutic regimens (**Figure 2**).

### 3.1 Induction of apoptosis

The activation of programmed cell death (apoptosis) is considered the major mechanism by which many essential oils have been shown to exert anticancer properties. The terpenoids (e.g. betulinic acid) (77) and phenylpropanoids (e.g. curcumin) (78) found in EOs can initiate and drive both the intrinsic and extrinsic pathways of apoptosis through Bcl-2 family protein interactions, initiation of caspase cascades, and modulation of death receptor signalling. The apoptosis induced by these compounds appears to be selectively triggered in tumor cells, as tumor cells typically exist in an environment with substantially higher levels of basal oxidative stress. It has been evidenced that EOs downregulate downstream genes such as Bcl-2, cyclin D1, and surviving (79) to reverse the anti-apoptotic and pro-proliferative programs. The fact that both pathways are activated provides a more aggressive means of inducing tumor regression and helps to circumvent the more common forms of resistance to apoptosis that frequently develop as a result of long-term chemotherapy treatment.

#### 3.1.1 Intrinsic mitochondrial pathway

EO constituents activate intrinsic mitochondrial pathways mainly through the reduction of mitochondrial membrane potential (ΔΨ_m_) (32, 80) and the subsequent release of cytochrome c from the inner membrane of mitochondria into the cytosol. β-Caryophyllene and limonene causes oxidative phosphorylation uncoupling and increases intracellular ROS (reactive oxidative species)concentration above sustainable apoptotic thresholds in cancer cells (81). The redox imbalance that occurs then facilitates the translocation of Bax and Bak pro-apoptotic proteins to the outer mitochondrial membrane (82), while at the same time decreasing the expression or activity of anti-apoptotic proteins such as Bcl-2 and Bcl-xL (83). Tumor mitochondria are thought to be particularly unique in their use of this pathway because they often have substantially less cardiolipin than normal mitochondria (84).

β-Caryophyllene and limonene increase ROS and mitochondrial dysfunction in cancer cells (85) via mechanisms resembling mild uncoupling or ETC disturbance. The structure and composition of cancer membranes are often different from those of normal mitochondria due to the incorporation of fat molecules into the membrane (86, 87). Both of these factors make tumor mitochondria particularly vulnerable to disruption of their membranes at the same time by lipophilic constituents of EOs. It also highlights how mitochondrial dysfunction creates further disruption of the electron transport chain complexes (88); this, along with the depletion of the levels of mitochondrially derived antioxidants, such as glutathione, increases in oxidative injury specific to cancer cells (89). Evidence from preclinical studies suggests that the mitochondrial dysfunction triggered by EOs promotes the activation of the apoptosome and effector caspases (90), which subsequently lead to a controlled dismantling of the cell without excessive inflammatory spillover. The effectiveness of this process can also be improved through the use of nanocarriers to deliver EOs (18), enhancing mitochondrial targeting and increasing the amount of time that EOs are retained in the cytoplasm of transformed cells.

#### 3.1.2 Extrinsic death receptor pathway

EO engage mechanisms of the extrinsic apoptosis pathway by upregulating the death receptors on cellular membranes and activating the death receptors such as the Fas (CD95) receptor, TRAIL-R1/DR4, and the caspase-8 activity (91). Concurrently, the receptors facilitate efficient transmission of apoptotic signals when the oils induce apoptosis through transductional signaling (92). For example, the extrinsic pathway requires a receptor-dependent assembly of the death-inducing signaling complex (DISC) that is responsible for activating initiator caspase-8 (93), which activates either the effector caspases directly or further amplifies this signal through the cleavage of Bid (94, 95). Concurrently, the pathway then also signals to the intrinsic pathway via bidirectional cross-talk to amplify apoptotic signals originating from factors present in the intrinsic mitochondrial pathway. Therefore, both pathways demonstrate sufficient apoptotic commitment to ensure that true apoptosis occurs. EO sensitization of cells to the death receptor ligands may be especially significant for cancers that express high levels of decoy death receptors (96) or have defects in components of the apoptotic signaling cascade downstream to the death receptors (97). Combination therapies utilizing EO constituents to restore or improve TRIAL signal transduction sensitivity to apoptosis (98, 99) suggest the possibility of developing synergistic therapeutic strategies that further optimize the pathway. Finally, by utilizing nanocarriers, target cell types can be provided with high localized concentrations of EOs at the membrane surface (18) to optimize apoptotic signaling while preserving the integrity of the volatile EO constituents and enhancing their intracellular retention.

### 3.2 Cell cycle arrest

EOs have been shown to produce a cell cycle block at either G1/S or G2/M checkpoints (100) due to their roles in prohibiting cell proliferation, providing an opportunity for cells to repair DNA or commit to apoptosis. Monoterpenes inhibits or modulates the expression and function of cyclin D1 and CDK4 (101), thus preventing the phosphorylation of retinoblastoma protein (Rb) and the subsequent release of E2F transcription factors from Rb, thereby promoting G1 phase arrest. Conversely, some sesquiterpenes (Parthenolide) can promote cell cycle arrest by blocking the formation of CDK1/cyclin B1 complex (34), which triggers G2/M checkpoint activation through activating p21WAF1/CIP1 (102). EO constituents also regulate checkpoint kinases [CHK1 and CHK2] (39) that phosphorylate Cdc25 phosphatases (39, 103), which prevent cells from entering mitosis. Earlier studies have shown that terpenoid treatment leads to increased levels of p53-independent p21 (104) in response to MAPK pathway mediated transcriptional activation, thus providing a mechanism for controlling the cell cycle in p53-deficient tumors. Similarly, EOs can prevent microtubule polymerization; for example, cinnamaldehyde binds to the β-tubulin subunit and induces the activation of the spindle assembly checkpoint (SAC) (105) and the extension of the metaphase period. Mitotic catastrophe, which has a different mechanism of action than the traditional apoptotic pathway, occurs as a result of microtubule destabilization induced by EOs. Studies assessing the effects of EOs on cellular metabolism have demonstrated EOs’ capacity to induce cellular growth arrest through G1 phase (106). This can be correlated with decreased rates of nucleotide synthesis, decreased rates of S phase progression, and, consequently, an overall restricted capacity for clonal expansion. The ability of normal cells to reverse their G1 arrest and re-enter the cell cycle following exposure to an EO (107) might have been contributing to the selective toxicities associated with EOs. Enhanced and sustained release of EOs through nanocarriers increases the duration of cell cycle arrest experienced following exposure to an EO and reduces the frequency of exposure and systemic peak concentrations required for effective treatments (**Table 1**). The combination of EOs with either CDK4/6 inhibitors or agents that target microtubules demonstrate synergistic effects in enforcing cell cycle checkpoint mechanisms (100) in breast cancer cell lines. EOs suppress tumor growth kinetics via the enforcement of cell cycle arrest at multiple nodal points and concurrently limit the activation of compensatory pathways. Modulation of multiple checkpoint pathways makes EOs an excellent option for adjunctive therapies in the development of additional treatment strategies targeting the cell cycle in the field of precision oncology.

**Table 1:** Summary of essential oil-based nanocarrier systems for anticancer activity.

Origin of essential oil	Encapsulation methods	Essential oil formulations	Carrier name	Major chemical compounds	Characterization of nanoparticles	Anti-Cancer Activity	Reference
Olive oil and Rosemary oil	Mini-emulsion methodology	QR-OO-NLC, QR-RO-NLC, QR-30RO3000-NLC	Nanostructured lipid carriers (NLCs)	Quercetin (QR); Olive oil phenolic compounds: hydroxytyrosol (HTyr), oleacein (ocein), oleocanthal (ocal); Rosemary oil compounds: carnosol, carnosic acid (CA), rosmarinic acid (RA)	Particle size: 177–199 nm; PDI: 0.446–0.557; ζ-potential: < −30 mV; antioxidant activity: 81–87%; cumulative skin permeation (24 h): 20.5–101.7 μg/cm².	Cytotoxic to A375 melanoma cells (QR-RO-NLC showed concentration-dependent cytotoxicity, whereas QR-OO-NLC was non-cytotoxic); enhanced intracellular/perinuclear and nuclear accumulation of quercetin; reduced melanoma burden with absence of superficial melanoma and fewer dermal tumor clusters; non-irritant in 3D skin model (cell viability >50%).	(108)
Algae oil 1; Fish oil; EPA oil; Olive oil	High-pressure homogenization (Panda Plus 2000, GEA Niro Soavi) at 500 to 600/100 bar; high shear mixing (Magic Lab, IKA-Werke) at 20,000 rpm	AOE1, AOE2, FOE, EOE, OOE	Nanoemulsion (oil-in-water)	AOE1: DHA (22:6 n-3) 67.7%, DPA (22:5 n-6) 14.3%, EPA (20:5 n-3) 0.6%; AOE2: EPA 34.9%, DHA 22.9%, DPA (22:5 n-3) 4.9%; EOE: EPA 84.9%, DHA 4.8%; FOE: EPA 19.5%, DHA 16.0%; OOE: OA (18:1 n-9) 71.0%, LA (18:2 n-6) 8.3%	AOE1: droplet size 268 nm, zeta potential -55.9 mV, pH 7.9; AOE2: 146 nm, -69.2 mV, pH 8.4; EOE: 182 nm, -48.8 mV, pH 8.0; FOE: 176 nm, -54.8 mV, pH 8.0; OOE: 367 nm, -61.0 mV, pH 7.6; all formulations: 100% <500 nm, conform to USP Chapter <729>	Cytotoxic to MUG Lucifer prim, MUG Lucifer met, and ESO-51 cells; AOE1 showed highest antitumor activity with no toxicity to normal fibroblasts; enhanced chemotherapy efficacy (synergistic with cisplatin); induced ferroptosis-related pathways and necrotic cell death.	(109)
*Rosmarinus officinalis* L.	Solvent injection-evaporation technique	Rosmarinus officinalis essential oil-loaded phytosomes	Phytosomes	Monoterpenes (e.g., 1,8-cineole, camphor, α-pinene); polyphenolic compounds (e.g., rosmarinic acid, carnosic acid, carnosol)	Particle size: ~223 nm; PDI: 0.22; ζ-potential: −53.9 mV; encapsulation efficiency: 80.1%; loading capacity: 9.4%; near-spherical nanoparticles; stable at 4 °C	IC50 against MCF-7: 99.4 μg/mL; IC50 against EA.hy926: 243.4 μg/mL; Selectivity Index: 2.45	(110)
Flaxseed (*Linum usitatissimum*) cold pressed oil	Phase-inversion temperature method	LF-PIP-FLX-LNC (lactoferrin-coated piperine and flaxseed oil-loaded lipid nanocapsules)	Lipid nanocapsules (LNC), called flaXosules	Piperine (PIP), flaxseed oil (FLX), lactoferrin (LF)	Particle size: ~77 nm; PDI: 0.20; ζ-potential: −2.0 mV; entrapment efficiency: 98.6%; sustained drug release (40.6% at 24 h); stable for 6 months; amorphous drug state confirmed	Potent anticancer activity against MDA-MB-231 cells (IC50 2.2 μg/mL); inhibited migration and markedly reduced tumor growth; induced apoptosis (↑caspase-3) and autophagy via AMPK/mTOR pathway modulation.	(111)
Copaiba oil (*Copaifera langsdorffii*)	Hot emulsion with sonication (HES)	oNLC, oNLC-Plu1%, oNLC-Chol0.05%, oNLC-Chi0.1%, oNLC-PEG1%	Nanostructured lipid carriers (NLCs)	β-caryophyllene (67.8%), α-humulene (9.6%), α-copaene (4.7%)	Particle size: 151–232 nm; PDI: 0.11–0.17; ζ-potential: −32.3 to +26.4 mV; entrapment efficiency: 97.2–99.9%; stable for 90 days at 4 °C	IC50 against MCF-7: oNLC 29.64 ± 0.55 μg/mL, oNLC-Plu1% 30.69 ± 0.70 μg/mL, oNLC-Chol0.05% 24.26 ± 0.70 μg/mL, oNLC-Chi0.1% 30.46 ± 0.72 μg/mL, oNLC-PEG1% 31.23 ± 0.52 μg/mL; CO alone 35.64 ± 0.44 μg/mL; oNLC-Chol0.05% showed 52.49% inhibition at 25 μg/mL	(112)
Kaffir lime (*Citrus hystrix*) leaf	Green synthesis (essential oil-mediated)	Kaffir lime essential oil-mediated silver nanoparticles (KLEO-AgNPs)	Silver nanoparticles (AgNPs)	Citronellal, citronellol, limonene, flavonoids, terpenoids, phenolic compounds	Particle size: 9–40 nm (SEM), 12–15 nm (TEM, mean 13.53 nm); Spherical, triangular, oval, and circular shapes; UV-Vis SPR peak at ~450 nm; EDX: Ag 88.13 wt%; ATR-FTIR confirmed involvement of O-H, C=O, C=N functional groups	IC50 against HeLa cells: 3.575 ppm; Dose-dependent cytotoxicity; Anti-apoptotic capability for cancer cells	(113)
Pistachio (*Pistacia vera*) *pericarp*	Green synthesis (essential oil-mediated)	Pistachio vera pericarp essential oil-mediated cerium oxide nanoparticles (PVEO-CeO2 NPs)	Cerium oxide nanoparticles (CeO2 NPs)	Not reported in the paper (GC-MS was performed per previous study but compounds not listed in this paper)	Pyramid-shaped, highly crystalline nanoparticles with cubic fluorite structure; average crystal size ~11.8 nm; phase-pure CeO_2_ confirmed by XRD	IC50 against LNCaP: 90 μg/mL (alone), 10 μg/mL (with ZA); IC50 against MCF-7: 40 μg/mL (alone), 5 μg/mL (with ZA); Dose- and time-dependent cytotoxicity; Synergistic effect with zoledronic acid (CI < 1); Upregulated BAX, downregulated BCL-2; Cell viability reduced to 78.13% (LNCaP) and 68.42% (MCF-7) with combination treatment	(114)
Clove (*Eugenia caryophyllata*) and Thyme (*Thymus vulgaris*)	Steam distillation followed by homogenization and ultrasonication (30 min, 350 W)	CL+TH-nanoemulsion (clove + thyme essential oil nanoemulsion)	Nanoemulsion	Eugenol (clove), Thymol (thyme)	Droplet size: 68.6 nm (DLS); PDI: 0.281; TEM: spherical morphology, size range 25.6-41.0 nm	IC50 values lower in MCF-7 than HepG2; CL+TH-nanoemulsion more cytotoxic than CL+TH-emulsion on both cell lines; CL+TH-nanoemulsion more effective and significantly cytotoxic than taxol on MCF-7; Increased activity of caspase-8 and caspase-9; Decreased VEGFR-2 activity in MCF-7	(115)
Frankincense (*Boswellia trees, family Burseraceae*)	Hydrotrope method (PEG 400 and P407)	HA/Gen-FO-Cub (hyaluronic acid-coated genistein and frankincense oil-loaded cubosomes)	Cubosomes (Cub)	Not reported in the paper (frankincense oil composition not listed)	Particle size: ~198 nm; PDI: 0.27; ζ-potential: −34.7 mV; entrapment efficiency: 99.3%; sustained drug release (43.7% at 120 h); spherical, monodisperse nanoparticles	Potent anti-pancreatic cancer activity (IC50 13.2 μg/mL); selective cytotoxicity with enhanced cellular uptake and antimigratory effect; significantly reduced tumor growth, NF-κB, VEGF, and Bcl-2 expression; increased tumor necrosis and antimetastatic activity	(116)
Macauba (*Acrocomia aculeata*)	Sonication method (315 W, 3 min x 3 cycles)	PM-MO (Macauba oil-loaded polymeric micelles)	Polymeric micelles (Pluronic® F-127)	Oleic acid (45.1%), linoleic acid (26.8%), palmitic acid (12.0%), β-carotene (2.81 mg/100g), phenolic compounds (632 mg/100g)	Size: 105 nm; PdI: 0.12; Zeta: -17.2 mV; EE: 80.51%; STable 6 months	Cytotoxicity against MDA-MB-231 (55% reduction at 386 μg/mL, 72h); No toxicity on L929; 97% colony reduction at 386 μg/mL; 40% migration inhibition at 48h	(117)
*Ocimum sanctum* (Tulsi)	Green synthesis (essential oil-mediated reduction of HAuCl_4_)	Os-AuNPs (Ocimum sanctum essential oil-gold nanoparticles)	Gold nanoparticles (AuNPs)	Methyl isoeugenol, caryophyllene, eugenol	Size: 57.81 nm (DLS), 24–28 nm (TEM); PDI: 0.42; Zeta: -27.6 mV; Spherical/triangular shape; SPR peak at ~540 nm	IC50 against MCF-7: 78.51 ± 2.4 μg/mL; G2/M phase arrest (10.3%); Increased ROS; Depolarized mitochondrial membrane; Annexin V positive; DNA fragmentation	(118)
Cinnamon (*Cynnamomum zeylanicum*), Sage (*Salvia sclarea*), Thyme (*Thymus vulgaris*)	Emulsification-ultrasonication method	CI-NLC, SA-NLC, TH-NLC	Nanostructured lipid carriers (NLCs)	Not reported in the paper	CI-NLC: 270.3 nm, PDI 0.272, ZP -27.8 mV; SA-NLC: 233.7 nm, PDI 0.227, ZP -27.8 mV; TH-NLC: 347.2 nm, PDI 0.110, ZP -33.4 mV; Stable for 12 months at 25 °C; Spherical morphology	IC50 against PC3 (48h): CI-NLC 0.156%, SA-NLC 0.180%, TH-NLC 0.019%; No toxicity to PNT2; Inhibited cell migration; Changed cell morphology; *In vivo*: reduced tumor size (TH-NLC 89.65% reduction) and angiogenesis	(119)
Geraniol (from essential oils of plants like *Monarda fistulosa*, palmarosa, *Thymus daenensis*, rose, citronella)	Homogenization with ultra-sonication method	NLC/GOH	Nanostructured lipid carriers (NLC)	Geraniol (GOH)	Size: 110 nm; PDI <0.2; Zeta: -10 mV; EE: 95%; STable 6 months at 4 °C; Spherical morphology	IC50 against A549: 1.0-1.5 mM range; 1.8-3.2 fold improved activity vs. free GOH; No toxicity to WI-38 fibroblasts; Increased MMP depolarization (34.5% loss); Increased cell death (19.2% at 1.0 mM); Reduced wound closure to 8.6% (vs. 65.7% control)	(120)
Bitter almond	Water/oil/water (W/O/W) dual nanoemulsion	Bitter almond oil as hydrophobic phase in dual emulsion	Graphene oxide (GO) nanocarrier coated with gelatin-polyvinylpyrrolidone (PVP-G-GO)	Quercetin (QC), bitter almond oil (amygdalin)	Size: 468 nm (PVP-G-GO-QC); Zeta: -40 mV; EE: 87.5%; Loading: 45%; Spherical morphology; pH-sensitive release (60% at pH 5.4 vs. 34% at pH 7.4 after 24h); Higuchi release model	Cell death against MCF-7: 53.14% (PVP-G-GO-QC), 36.51% in apoptotic phase; Biocompatible on L929 (92% viability); Free QC: 48.84% cell death	(121)
Thyme (*Thymus vulgaris* L.)	Ionotropic gelation with STPP crosslinking	TOE-BPE-CSNPs (ternary nanoformulation with bee pollen extract)	Chitosan nanoparticles (CSNPs)	Thymol (8.32%), carvacrol (6.96%), germacrene D (10.10%), α-acorenol (6.25%)	Size: 51.76-109.21 nm; PDI: 0.21-0.31; Zeta: +46.28 mV; EE: 92.1% (TOE), 77.2% (BPE); Spherical morphology	IC50 against HepG2: 60.2 μg/mL; IC50 against MCF-7: 55.6 μg/mL; No toxicity to Vero cells (IC50 >86.5 μg/mL); Apoptosis: 49.3% (HepG2), 76.1% (MCF-7); Upregulated caspase-3 (9-fold in MCF-7) and P53; Decreased TNF-α and IL-6 (p<0.001)	(122)
*Pistacia lentiscus* Var. Chia	Thin-film hydration method	PO/NS (Pistacia lentiscus oil-loaded niosomes)	Niosomes (Span 60, Tween 60, cholesterol)	α-Pinene (81.20%), β-Myrcene (4.70%), β-Pinene (2.97%), Linalool (1.51%)	Size: 112.38 ± 13.1 nm; PDI: 0.13 ± 0.06; Zeta: -13.09 ± 2.9 mV; EE: 93.4 ± 15.1%; Sustained release (86.8 ± 6.1% at 72h); STable 21 days	IC50: Skov-3 4.88 μg/mL, MCF-7 7.39 μg/mL (>200 μg/mL on MCF10A); 10-fold increased cytotoxicity vs. free PO; Combined apoptosis: 21.19% (Skov-3), 14.33% (MCF-7); Sub-G1 arrest; Upregulated Bak/Bax, downregulated Bcl-2	(123)
Piper chaudocanum leaves (Son La Province, Vietnam)	Green synthesis (essential oil-mediated reduction of AgNO_3_)	P. chaudocanum essential oil-mediated silver nanoparticles	Silver nanoparticles (AgNPs)	Nerolidol (73.56%), spathulenol (3.45%), caryophyllene oxide (2.63%), farnesol (1.20%)	SPR peak at 430 nm; XRD: FCC crystal structure, size ~14 nm; SEM: spherical morphology; FTIR confirmed functional groups	IC50 against HepG2: 13.8 μg/mL (AgNPs), 22.9 μg/mL (essential oil); Dose-dependent cell death (36.31% mortality at 10 μg/mL AgNPs vs. 12.49% for oil); Morphological changes (circular shape, membrane shrinkage, vacuolization)	(123)
Cinnamon cassia (*Cinnamomum cassia* Presl) bark	Ionic crosslinking method	CS-CEO (cinnamon essential oil-loaded chitosan nanoparticles)	Chitosan nanoparticles	trans-Cinnamaldehyde (39.64%) (–),-Copeane (17.26%), (+)-delta-Cadinene (11.17%), alpha-Muurolene (7.70%)	Particle size: ~215 nm; PDI: 0.246; ζ-potential: +51.7 mV; encapsulation efficiency: 83.4%; drug loading: 26.4%; spherical nanoparticles; stable for 60 days at 4 °C	Enhanced anticancer activity against MDA-MB-231 cells; inhibited migration and invasion; induced oxidative stress, mitochondrial dysfunction, and apoptosis (↑caspase-3, AIF); significantly suppressed tumor growth and proliferation *in vivo*	(124)
*Salvia officinalis* L. (Sage)	Solvent diffusion technique	FPSP/EO/silver nanoparticles, PSP/silver/EO	PLA-Spermine-PEG-FA (FPSP) copolymer nanoparticles	β-thujone, α-thujone, camphor, 1,8-cineole	Size: 200–300 nm (FPSP/silver/EO ~249 nm); Zeta: -20.6 ± 1.3 mV (FPSP/silver/EO), -18.3 ± 0.7 mV (PSP/silver/EO); Spherical morphology; Good monodispersity	IC50 against AGS: 21.11 μL/mL at 72h (FPSP/silver/EO); No toxicity to GES-1 normal cells; Minimum cell viability 17.04% at 50 μL/mL (48h); FPSP/silver/EO better than EO and PSP/silver/EO	(125)
Red Dragon (*Hydrocerus polyrhizus*) pulp and seed oil	Green synthesis (essential oil-mediated reduction of HAuCl_4_)	AuNPs@D.pulp_seed oil	Gold nanoparticles (AuNPs)	Oleic acid (30.94%), stearic acid (17.48%), palmitic acid (16.59%), flavonoids, phenolic compounds, carotenoids, organic acids	Size: 25.31 nm (XRD), 36.2 nm (DLS), 8.65-36.2 nm (TEM); Zeta: 18.4 mV; SPR peak at 540 nm; Spherical morphology; FCC crystal structure	IC50 against HCT-116: 100 μg/mL, HepG2: 155 μg/mL, MCF-7: 165 μg/mL; 92.89% inhibition against HCT-116 at 500 μg/mL; Dose-dependent cytotoxicity	(126)
Greater celandine (*Chelidonium majus* L.) roots and leaves	Emulsion-ionic gelation method	CNPs-GCREO (roots), CNPs-GCLEO (leaves)	Chitosan nanoparticles (CNPs)	(E) Anethole (44.62-51.04%), limonene (17.83-22.39%), α-pinene (3.84-18.38%)	Size: 76.5-115.3 nm; PDI: 0.179-0.225; Zeta: +34.3 to +37.1 mV; EE: 62.5% (roots), 69.1% (leaves); Spherical morphology	IC50: GCLEO 90.2, GCREO 126.4, CNPs-GCREO 77.6, CNPs-GCLEO 41.5 μg/mL; Apoptosis: CNPs-GCLEO 63.73%; CNPs non-toxic (96.43% viability at 320 μg/mL)	(127)
*Juniperus squamata* roots	π-π/hydrophobic interactions	GO-PVP-JSEO	Graphene oxide functionalized with polyvinylpyrrolidone (GO-PVP)	α-Chenopodiol (5.51%), Terpinene-4-ol (5.38%), Cadin-4-en-10-ol (5.36%) (–),-Thujopsen (5.30%)	Raman ID/IG: GO 0.84, GO-PVP 0.85, GO-PVP-JSEO 0.86; FTIR confirmed functionalization; XRD: GO peak shifted from 10.99° to 7.66°; JSEO loading capacity: 17.4% (GO-PVP) vs. 13.6% (GO)	IC50 against MDA-MB-231: 4.04 ± 0.87 μg/mL (GO-PVP-JSEO) vs. >20 μg/mL (JSEO); Cell survival at 20 μg/mL after 72h: JSEO 64%, GO-JSEO 29%, GO-PVP-JSEO 21%; No significant toxicity to HEK293T	(128)
*Pistacia atlantica* Desf. gums	Probe ultrasonication method	PAEO-NLC4	Nanostructured lipid carriers (NLCs)	α-Pinene (>95%), D-limonene	Size: 151 ± 1 nm; PDI: 0.16 ± 0.03; Zeta: -29.1 ± 1.4 mV; EE: 99.3%; LC: 9.6%; Spherical morphology (TEM)	IC50 against SKBR3: 0.08 μg/mL (24h), 0.09 μg/mL (48h); Increased Sub-G1 population (27.13% at 48h vs. 5.66% control); Induced apoptosis; S phase arrest	(129)
*Artemisia vulgaris* L.	Single emulsion solvent evaporation method (W/O) with CS-FA coating	AVEO-PCF-NPs	PLGA nanoparticles modified with chitosan-folic acid	Not reported in the paper	Size: 298.96 nm; PDI: 0.055; Zeta: +20.32 mV; EE: 91.8%; FA binding: 65%; Spherical morphology	IC50 against HT-29: ~78 μg/mL; No toxicity to HFF normal cells; SubG1 arrest; AO/PI staining confirmed apoptosis; CAM assay showed anti-angiogenesis	(130)
Eugenol and vanillin are essential oil components	Covalent immobilization via alkoxysilane derivatives	Eugenol-functionalized SAS, MCM-41 micro, MCM-41 nano; Vanillin-functionalized SAS, MCM-41 micro, MCM-41 nano	Silica particles (SAS, MCM-41 micro, MCM-41 nano)	Eugenol, vanillin	Size (TEM): SAS 2.24 μm, MCM-41 micro 1.22 μm, MCM-41 nano 73.7 nm; Zeta potential: negative (bare) to positive (functionalized); Functionalized MCM-41 micro most cytotoxic (IC50 0.17-0.19 mg/mL at 48h)	IC50 against HepG2: eugenol 1.27-1.29 mM, vanillin 2.53-2.87 mM; Functionalized particles more cytotoxic than free EOCs; Functionalized MCM-41 micro most potent (IC50 ~0.17-0.24 mg/mL)	(131)
Olive oil (purified)	Solvent-displacement technique	αCD44-O²LNC (anti-CD44 antibody-conjugated olive oil liquid nanocapsules)	Olive oil liquid nanocapsules (O²LNC)	Olive oil triglycerides	Size: 111 ± 18 nm; PDI: ~0.10; Zeta: -35 mV (αCD44-O²LNC at pH 7.4); Antibody coupling efficiency: 93.4%; DL: 2.2%; EE: 81.1%	IC50 against pancreatic CSCs (BxPC-3 PCSCs): αCD44-O²LNC-PTX 34.2 nM (4-fold vs. free PTX 133.4 nM); Selective uptake in CD44^+^ cells; *In vivo* tumor targeting confirmed	(132)
Clove (*Syzygium aromaticum*)	Ultrasonic homogenization	NE-CLV (clove oil nanoemulsion), IF-CLV (ifosfamide-loaded clove oil nanoemulsion)	Nanoemulsion (O/W)	Eugenol (clove oil)	NE-CLV: size 63.1 ± 1.00 nm, zeta -4.39 ± 0.4 mV; IF-CLV: size 89.4 ± 2.64 nm, zeta -11.65 ± 1.1 mV; PDI <0.05	IC50 against HeLa: NE-CLV 0.20 mM, IF-CLV 0.13 mM (57-fold vs. free IF 7.69 mM); IC50 against MCF-7: NE-CLV 0.34 mM, IF-CLV 0.26 mM (35-fold vs. free IF 9.20 mM); Dose-dependent cytotoxicity; Apoptosis confirmed by DAPI staining	(133)
Green tea (*Camellia sinensis*)	Emulsion followed by ionic cross-linking with TPP	CS/GTO NPs	Chitosan nanoparticles (CS NPs)	Catechins (–),-epigallocatechin-3-gallate (EGCG)	Size: 30.7 ± 1.13 nm (TEM), 256.3 ± 6.5 nm (DLS); Zeta: 29.0 ± 0.2 mV; EE: 81.4 ± 5.7% (UV-Vis), 85.98 ± 1% (TGA); Spherical morphology	IC50 against HepG2: 61.5 ± 1.4 μg/mL (3-fold vs. CS NPs), MCF-7: 96.9 ± 3.5 μg/mL (2.3-fold), HCT-116: 108 ± 4.3 μg/mL (1.7-fold); 99mTc-labeled with 93.4 ± 1.2% RE; Tumor uptake 20.3 ± 2.1% at 30 min; T/NT ratio 7.8 at 0.5h	(134)
*Zataria multiflora*	Emulsification with tween-20 (mild emulsion conditions)	CP/ZEO NE (Citrus pectin nanoemulsion of Zataria essential oil)	Citrus pectin (CP) nanoemulsion	Thymol, carvacrol	Size: 165 ± 6 nm (at 100 μg ZEO); Zeta: -40 ± 2.5 mV; FTIR confirmed non-covalent interactions; Stable for 120 days	IC50 against MDA-MB-231: 20.4 μg/mL (72h), T47D: 0.0016 μg/mL (72h), MCF-7: 5.38 μg/mL (72h); No toxicity to L929; Induced ROS, MMP loss, G2/M arrest, DNA damage (comet/TUNEL); 38% apoptosis at 10 μg/mL	(135)
Morinda citrifolia seeds	Ionic gelation with TPP crosslinking	MCEOs-CHs NPs	Chitosan nanoparticles (CHs NPs)	L-Scopoletin, nordamnacanthal, β-morindone, α-copaene, β-thujene, terpinolene	Size: 1006 nm (DLS), Zeta: 43.5 mV; PDI: 0.815; FTIR confirmed interactions; SEM/TEM showed spherical morphology	IC50 against A549: 40 μg/mL (54% inhibition); AO/EB showed apoptosis; Hoechst 33342 confirmed nuclear damage; ROS increased; Mitochondrial membrane potential loss; G1 phase arrest (56.27% vs. 36.45% control); Hemolysis 2.4%	(136)
*Zataria multiflora*	Mild emulsification with TWEEN 20	CS/ZEONE	Chitosan nanoparticles (CS NPs)	Thymol, carvacrol	CS: size 305 ± 16 nm, zeta +25.27 ± 2.0 mV; CS/ZEONE: size 463 ± 18 nm, zeta +18.35 ± 1.7 mV; FTIR confirmed non-covalent interactions	IC50 against MDA-MB-231: 6.2 μg/mL (72h), T47D: 1.4 μg/mL (48h), MCF-7: 56 μg/mL (72h); No toxicity to L929; Sub-G1 accumulation (27.29% at 12 μg/mL, 24h); G2/M arrest; ROS increased 39-fold; MMP loss 59%; DNA fragmentation; 8-oxo-dG increased	(137)

### 3.3 Inhibition of angiogenesis in tumors

Growth factors like VEGF (138) and FGF-mediated tumor-induced angiogenesis is negatively affected by the multiple mechanisms by which EOs (139) interfere with both endothelial cell function and pro-angiogenic gene expression. For example, terpenoid compounds such as perillyl alcohol (140) and eucalyptol decrease hypoxic stabilization of HIF-1α (141), thereby possibly limiting transcription and secretion of VEGF. Additionally, phenylpropanoid compounds such as eugenol prevent phosphorylation of VEGFR-2 (142), leading to impaired activation of downstream ERK, reducing endothelial cell migration and proliferation. Consistent with these *in vitro* results, EO-treated human umbilical venous endothelial cells demonstrate disrupted capillary network assembly with lower branching density in tube formation assays (143). Mechanistically, EOs inhibit secretion of MMP-2 and MMP-9 (144), limiting the degradation of extracellular matrix required for vascular sprouting. In addition to the anti-angiogenic effects of EOs, β-caryophyllene has demonstrated anti-angiogenic activity mainly by modulating VEGF signaling in preclinical models (145). It has been shown that the use of EOs may help to reduce blood vessel formation and improve blood flow to cancerous tissues. Researchers found that using these oils led to lower levels of thrombosis (146) and that the use of these oils increased levels of thrombosis inhibiting substances. EOs thus contribute to preventing or treating cancerous tissues by inhibiting the formation of new blood vessels.

### 3.4 Suppression of metastasis and epithelial–mesenchymal transition

Emerging studies suggest that EOs have been shown to significantly reduce metastasis of cancer cells through the inhibition of epithelial-mesenchymal transition (EMT) (147). EMT is a normal process that occurs during embryonic development, but it is also appropriated by carcinomas to gain the ability to migrate and invade. Researchers showed that the constituents of EOs promote expression of epithelial markers, such as E-cadherin and ZO-1 (148) while simultaneously suppressing mesenchymal transcription factors such as Snail, Slug, Twist, and ZEB1 (147), thereby restoring cell-to-cell adhesion and polarity. Furthermore, terpenoids, such as α-pinene and limonene have been shown to inhibit the TGF-β/Smad signaling pathway (149, 150). Therefore, in cancer the same phenomenon could drives EMT by increasing the expression of Smad7 and preventing the activation of the receptor kinases (147). Phenylpropanoids, such as chavicol, inhibit the phosphorylation of focal adhesion kinase (FAK) and Src (151), disrupting cytoskeletal reorganization and the formation of lamellipodia, the cellular structures necessary for cell migration. *In vitro* assays that utilize wound healing and transwell chambers show that the metastatic cell lines treated with EOs have a reduced ability to migrate from the site of treatment (147).

In addition, the use of EO reduces the activity of matrix metalloproteinases (MMP); specifically, MMP-2, MMP-9 and MMP-14 (152) to limit degradation of the basement membrane and extravasation of tumor cells. EOs also inhibit the ability of the CXCR4/CXCL12 chemokine signaling pathway (153); thereby limiting the homing of tumors to the microenvironments of bone, liver, and lung. Proteomic analysis of cells treated with EOs showed that EOs increased the expression of miR-200 family members that directly target ZEB1/2 mRNA (154). This establishes a feedback loop that maintains and stabilizes epithelial differentiation. Additionally, in preclinical animal models, tumor growth in the lung and liver significantly decreased after administration of EOs (155), either prophylactically or therapeutically. Finally, it is also observed that EO-derived compounds sensitize cancer cells to anoikis and reduce survival of aggressive metastatic cells in preclinical models, partly by interfering with integrin/FAK signaling (156). Through preventing the initiation of EMT and subsequent migration, invasion and colonization by the tumor, EOs represent an innovative, comprehensive anti-metastatic approach that targets multiple steps during the metastatic cascade. Furthermore, it is possible that nano-delivery systems could increase the anti-metastatic activity of EOs by sustaining EO concentrations at invasive fronts and at the lymphatic interface.

### 3.5 Modulation of inflammation and immune response

Currently evidences are emerging where chronic inflammation is seen as a contributor to cancer development (157). EOs are effective at regulating the immune system by balancing the levels of two different types of inflammation signals [pro-inflammatory (158) and anti-inflammatory (159)] present in the area surrounding a tumor. Some of the components found in EOs prevent or inhibit the transcription of IL-6, IL-1β, TNF-α, and COX-2 (160, 161) which could reduce the ability for macrophages to undergo M1 polarization and create neutrophil extracellular traps (NETs) that promote tumor growth. However, certain terpenes can help drive the polarization of macrophages from M2 to M1 (162) and enhance their ability to phagocytose dead cells and present antigens (163). Report suggests that in general β-Caryophyllene acts at the CB2 receptor and prevents the activation of the NLRP3 inflammasome contributing towards reduced tissue damage and immunosuppression associated with pyroptosis (164). The ability of isoeugenol (165) and linalool (166) to enhance dendritic cell maturation results in an increase in MHC-II and CD80/86 expression (167). This leads to more effective priming of T-cells as well as increased infiltration of the cytotoxic lymphocytes. The JAK/STAT axis can be modulated by EOs (168) which could be lead to a T_H_1-dominant immune response to counteract the growth of T-regulatory cells induced by tumors. Studies have shown that volatile phenolic compounds in EOs could also inhibit IDO1 (169) preserving available tryptophan and averting T-cell anergy. Additionally, EOs administered to mice showed an increase in CD8^+^ TIL density (170) and a decrease in PD-1 and PD-L1 protein expression (171), suggesting EOs can cooperate with immune checkpoint blockade therapy. Importantly, EOs modulate the immune system in a concentration-dependent manner (172). Furthermore, studies have shown that nanoencapsulation protects the immune-modulating fraction of the EO (173) from rapid hepatic clearance, allowing long-term reprogramming of TAMs (174).

### 3.6 Oxidative stress and antioxidant modulation

EO exhibit a contradictory ability to modulate redox status; they act as pro-oxidants in cancer cells but have antioxidant properties in normal tissues (8). This dichotomy is due to the high basal level of ROS in cancer cells and their deficient levels of antioxidant enzymes. Limonene and α-pinene induce mitochondrial ROS in cancer cells by disrupting the electron transport chain and oxidative phosphorylation, promoting apoptosis (175). In addition, EO components deplete glutathione (GSH) stores by conjugating with electrophilic metabolites and inhibiting the activities of glutathione peroxidase and thioredoxin reductase (176). The resulting redox imbalance damages DNA repair enzymes and activates p53-independent stress-response pathways. On the other hand, phenylpropanoids, such as eugenol and thymol, exhibit radical-scavenging properties in healthy cells by donating a hydroxyl group and chelating metals (177). Further, this induces the upregulation of Nrf2/ARE pathways to increase the expression of SOD (superoxide dismutase), catalase, and HO-1 (heme oxygenase-1). The localized use of redox modulation limits the systemic extent of oxidative harm and provides the maximum amount of cytotoxicity for the tumor. Metabolomic studies have shown that exposure to EOs affects the ratio of NAD^+^/NADH in cells disrupting survival signalling by sirtuins and producing metabolic stress (178). Nanoparticle formulations have been designed to target EO redox-active fractions directly into the mitochondria of tumors (179) for the purpose of magnifying local ROS fluxes while protecting the systemic circulation. EO synergize with radiotherapy and platinum chemotherapy by depleting GSH, increasing oxidative injury and impairing detoxification of DNA-damaging agents in cancer cells (180). On the other hand, EO-based antioxidants could prevent injury to heart or kidney following chemotherapy, thereby improving the therapeutic index. Through targeting the redox vulnerability of cancer cells, while maintaining homeostasis of normal tissue, EO represent an approach of precision redox medicine that maximizes the efficacy of therapy while minimizing the collateral damage to healthy tissues.

### 3.7 Selective cytotoxicity of EO

The selective cytotoxicity of EOs against cancer cells relative to normal healthy cells represents a fundamental attribute that underpins their therapeutic potential. This selectivity arises from multiple convergent mechanisms that exploit the distinct biochemical and biophysical properties of malignant cells. Cancer cells exhibit intrinsically higher basal levels of reactive oxygen species (ROS) due to dysregulated metabolism and mitochondrial dysfunction, rendering them more susceptible to the pro-oxidant effects of EO constituents (8, 32). Terpenoids such as limonene and α-pinene induce mitochondrial ROS generation by disrupting the electron transport chain, pushing cancer cells beyond their already-elevated oxidative threshold into apoptotic commitment, while normal cells with robust antioxidant reserves remain protected (175).

The differential lipid composition of cancer cell membranes further contributes to selective vulnerability. Tumor cells incorporate altered lipid profiles with reduced cardiolipin content in mitochondrial membranes, making them more susceptible to disruption by lipophilic EO constituents (84, 87). This structural difference facilitates preferential uptake and membrane perturbation in malignant cells. Preclinical studies have consistently demonstrated that EOs induce dose-dependent cytotoxicity in cancer cell lines while sparing normal epithelial cells. For instance, Carvone, the major monoterpene in spearmint oil, selectively inhibited the proliferation of MDA-MB-231 and MCF-7 breast cancer cells (IC_50_ ≈1.0 and 1.2 mM, respectively) with lower toxicity toward healthy MCF-10A cells. Its anticancer activity involved ROS-induced DNA damage, S-phase arrest (in MCF-7), and p53- and caspase-mediated apoptosis, marked by upregulation of p53, Bad, cleaved caspase-3, and cleaved PARP (181).

Additionally, EO constituents such as geraniol have demonstrated selective suppression of MCF-7 cells by inducing G1 phase cell cycle arrest through inhibition of cyclin D1, E, and A as well as CDK2 and CDK4, while showing minimal effects on non-malignant cells (107). This selectivity could be further enhanced through nanoformulation strategies that enable targeted delivery and preferential accumulation at tumor sites, thereby widening the therapeutic window (18). Studies evaluating the selectivity index (SI) of EO formulations consistently report SI values >2.0, indicating preferential toxicity toward cancer cells compared to normal fibroblasts or epithelial cells (5, 9).

## 4 Key signalling pathways modulated by EO

EO act as anticancer agents via direct interference in the abnormal signalling pathways that enable the survival, proliferation, and immune evasion of tumor cells (8, 32, 180). These oils can interact with and inhibit multiple targets; they are essentially multi-modulators that can simultaneously impact several different signalling pathways, including kinase signalling cascades (182), transcription factors (183) and epigenetic regulators (184). The effect of EOs on the simultaneous modulation of multiple molecular targets results in the systems-level disruption of oncogenic signalling that limits or delays compensatory activation of alternative pathways and delays the acquisition of resistance to therapy. Understanding the molecular dynamics of EOs is paramount to developing EOs as adjunct therapies for existing targeted therapies in the context of precision oncology.

### 4.1 NF-κB pathway

A key part of inflammatory signalling, regulation of cell viability, and regulation of responsiveness to chemotherapeutic agents, the NF-κB pathway is often hyperactivated in diverse tumors (185). Evidence states that EOs obstruct this pathway through multiple levels beginning with inhibition of the activation of the IκB kinase (IKK) complex (186). For example, terpenes such as β-caryophyllene and limonene inhibit phosphorylation of IKKβ (187–189). This leads to stabilization of IκBα in the cytoplasm and retention of NF-κB p65/p50 dimers in their inactive form (190, 191). Further upstream, terpenoids inhibit nuclear transport (192) by blocking importin-α/β (193) interactions and facilitates CRM1-mediated nuclear export of NF-κB dimers (194). Additionally, some monoterpenes (e.g., carvone) modulate NF-κB p65 acetylation through CBP/p300 pathways (195). However, HAT inhibition by EO compounds (carvone) is generally indirect or context-dependent (195). EOs also modulate the upstream TLR4/MyD88 signalling pathway (196), thereby reducing the recognition of pathogen-associated molecular patterns (PAMP) and damage-associated molecular patterns (DAMP) to maintain chronic NF-κB activation. Moreover *Eucalyptus* oil inhibits NF-κB in macrophages associated with cancer by releasing cytokines from TNF-α, IL-6, NO, iNOS and COX-2 (197), which leads to a normalization of the microenvironment. The combined use of an IκK inhibitor and/or proteasome modulators (198) provides synergistic inhibition of NF-κB, but especially for multiple myeloma (198) and pancreatic adenocarcinoma (8, 199). Destroying this major survival switch could restore the apoptotic potential of resistant cancers. Furthermore, it could also make them sensitive to standard treatment, effectively establishing the inhibition of NF-κB as a primary mechanism for successful treatment in precision phytotherapy. Recently, via nanoencapsulated oil fractions, there is more efficient delivery to the nucleus sustained for a longer period of time at lower systemic concentrations (200, 201).

### 4.2 PI3K/AKT/mTOR axis

The PI3K/AKT/mTOR pathway is a major regulator of cell growth, metabolism, survival, and autophagy and is one of the most commonly dysregulated signalling pathways in many types of human cancer (202). Preclinical studies have shown that the constituents of EOs provide multi-point inhibition of this pathway through decreased PI3K enzyme activity (203), phosphorylation of AKT at regulatory sites—Ser473, Thr308 (204); mTOR signalling (205), and decreased effects of the mTOR pathway on downstream effectors S6K1 (206) and 4E-BP1 (207). Some constituents of EOs may also affect the pathway through ROS-mediated crosstalk (208), or support the functional integrity of the tumor suppressor PTEN (209). This further, functions as an important negative feedback regulator of PI3K through the dephosphorylation of PIP3, leading to a decrease in PI3K-dependent signalling (210, 211). As per reports, EOs reduce cell survival signals (32), decrease protein synthesis (212), interfere with metabolic reprogramming (213), and promote apoptosis (32) in cancer cells. Recently, Anwar et al. (214), have shown that EO derived compound p‐cymene has 216 common targets between p‐cymene and hepatocellular carcinoma. Among these, target PSMD12 have been found to be one of the major protein that could that acts like an accelerator for liver cancer by helping cancer cells grow, multiply, and survive (215). The above has been seen in several cancer models containing PI3K and/or AKT oncogenes (216). Administration of EO via nanoparticles could enhance the inhibition of signalling pathways by providing more intracellular delivery. The use of both synthetic PI3K and AKT inhibitors and EO constituents could demonstrate an additive or synergistic response in many instances, and could act as adjunct modulators in rational combination therapy. Continued study of the contribution of individual EO constituents is needed to better understand them and develop more effective clinical applications.

### 4.3 MAPK/ERK and JNK pathways

The MAPK signalling pathway, which includes ERK, JNK, and p38 pathways is responsible for regulating cell division (217), responding to stress, and inducing apoptosis (programmed cell death). Disruption of MAPK signalling pathway can contribute to the development of cancer (218). EOs contain some of the same compounds as other drugs or chemicals that affect one or more of the three MAPK pathways or signal cascades. For example, limonene and linalool are both terpenoids that block phosphorylation of RAF-MEK-ERK by disrupting scaffolding proteins at the plasma membrane (219). This decreases the amount of transcription for c-Myc and cyclin D1. Evidence suggests that cinnamaldehyde derivatives can activate JNK1/2 by phosphorylating ASK1 and recruiting MKK4/7 to JNK (220). This activation of JNK promotes activation of c-Jun and ultimately expression of pro-apoptotic genes (221). By modulating different pathways in opposing ways, EOs could create an imbalance in signalling that favors cell death over division. Additionally, proteomic studies indicate that EOs reduce the phosphorylation of BAD through ERK signaling (222), thus releasing BAD to bind Bcl-2 at the mitochondria (223). In addition, the exposure to EO increases the production of dual-specificity phosphatases (DUSP) (224), which dephosphorylate, and subsequently, deactivate ERK, causing a negative feedback loop that limits compensatory re-activation. Subsequently, in models of oxidative stress, compounds found in EOs activate JNK and promote phosphorylation of p53 at serine 392 (225), leading to greater transcriptional activity toward pro-apoptotic proteins, Bax and PUMA. Furthermore, the use of nanoencapsulation would serve to enhance JNK activation by maintaining intracellular levels of EOs greater than those needed to engage the kinase. The combination of MEK inhibitors or JNK modulators provides a synergistic control of the signalling pathways, especially in RAS mutants (226).

### 4.4 JAK/STAT3 signalling

Constitutive phosphorylation of JAK/STAT3-mediated cytokine (IL-6) is widely linked to proliferation, evasion from immune attacks and maintaining stemness, indicative of poor outcomes in several cancers (227). EOs have been studied to determine their effects on this signalling pathway. Several bioactive constituents of EOs act at multiple levels on JAK/STAT3 signaling system by directly inhibiting the activity of the JAK2 kinase, the effector of this pathway (228). erpenoids such as β-caryophyllene exhibit anti-inflammatory effects and can modulate JAK/STAT signaling pathways (229), phosphorylating STAT3 at Tyr705 (230). Thalappil et al. (79), have shown that EOs from *Pinus mugo*, *Lavandula angustifoglia*, *Pinus sylvestris*, and *Cupressus sempervirens* could prevent phosphorylation of STAT3 at Tyr705. Till date, there is no evidence of EO or its derivatives that could particularly, inhibit SH2 domain binding of STAT3 dimeric complexes. This induces SOCS3 expression, a natural feedback inhibitor capable of suppressing STAT3 phosphor dimer formation (231, 232). The effects of EOs at the gene level also include; decreased expression of survivin, Bcl-xL, and VEGF (233), leading to a reversal of pro-survival expression programs. In addition, *in silico* approaches confirm that carvacrol could possibly induce changes in the acetylation by p300 and increases STAT3 nuclear retention (234). Thus, it could lead towards enhancing the affinity of STAT3 with DNA at the promoters of γ-activated sequences (GAS) (235). Furthermore, the component of EOs inhibit the upstream signalling of IL-6 (197) and inhibit Src/STAT3 signalling to reduce small-cell lung cancer cell growth and survival (236). This results in reduced numbers of ALDH1^+^ and CD44^+^ cancer stem cells (237) leading to decreased self-renewal and resistance to chemotherapy. The use of EOs in conjunction with either JAK Inhibitors or STAT3 decoy oligonucleotides produces additive inhibition of the signalling pathways; especially in the case of triple negative breast cancer and hepatocellular carcinoma. EOs, therefore, could act as a master regulator of the crosstalk between tumors and immune system, restoring immunological surveillance, sensitizing resistant clones and providing a rational approach for precision phytotherapy targeted at cytokine-mediated oncogenesis.

## 5 Pharmacokinetic challenges and limitations

EOs have potential anticancer effects however, they encounter several pharmacokinetic barriers which limit their usefulness in clinical practice (238). These barriers include high lipophilicity (239), volatile nature (8) and oxidative degradation (240) that leads to poor water solubility (241), fast systemic clearance, and variable bioavailability (18). To improve their stability, increase circulation time, and achieve therapeutic levels at tumor sites, more sophisticated formulations are needed which are discussed further.

### 5.1 Poor aqueous solubility and stability

EOs have very low solubility in water, which limits their ability to dissolve and become available for absorption into the bloodstream (239, 241). Furthermore, due to their high volatility (8), monoterpenes readily evaporate and/or oxidize when exposed to heat, light or oxygen, causing them to degrade into less-active and/or potentially toxic degradation products. In addition to being unstable due to their volatility, these oils are also highly sensitive to pH (242) and therefore can be chemically unstable when exposed to acidic environments. Furthermore, the non-polar portion of the chemical compound (terpene) will migrate rapidly out of the biological matrix. This could be through partitioning into lipid membranes or binding to plasma proteins, resulting in very low levels of free drug available for targeting to a tumor. Due to the physicochemical limitations of EOs, the challenges associated with achieving a reproducible dose, short shelf-life, and complex formulations can be addressed by using a variety of stabilization techniques, including encapsulation in a hydrophilic polymeric matrix or an amphiphilic carrier to provide a protective environment for volatile compounds, improving their ability to disperse in an aqueous environment and minimising their oxidative instability (242) when stored. Stabilized formulations with improved pharmacokinetics provide greater consistency and allow for reliable preclinical evaluation for clinical efficacy.

### 5.2 Low oral bioavailability and rapid metabolism

First-pass metabolism and intestinal efflux mechanisms severely limit the oral administration of EOs (243). Because of the differences in xenobiotic handling, terpenoid constituents of EOs are extensively oxidized by phase 1 oxidation through CYP450 forms (i.e., CYP3A4 and CYP2C9) and conjugated by phase 2 processes (i.e., glucuronidation and sulfation) resulting in highly diminished systemic exposure (17). The efflux of unmetabolized EO fractions from enterocytes to the intestinal lumen occurs via P-glycoprotein and BCRP transporters, which are the primary causes of diminished absorption (244). For most major monoterpenes in humans, there is high hepatic clearance rates, ~70% pulmonary uptake but rapid systemic metabolism (245). This may force the use of excessive dosing frequencies and create risks for gastrointestinal irritation and/or hepatotoxicity. Rapid metabolism produces differing active metabolite profiles among individuals, creating unpredictability in pharmacodynamic responses. Potential strategies to address these limitations include prodrug derivatization, inhibiting transporters, and bypassing gastrointestinal degradation via alternative delivery routes. Currently there is ongoing interest with using nanocarrier-mediated protection from enzymatic hydrolysis and utilizing targeted lymphatic uptake of EOs for providing improvement of their systemic bioavailability to provide sustained therapeutic concentrations with reduced dosing requirements.

### 5.3 Off-target toxicity and dose optimization

Though EOs possess selective cytotoxicity, they may also cause off-target effects such as hepatotoxicity, neurotoxicity, and irritation of the gastrointestinal mucosa (246, 247), when administered at high systemic concentrations. Dose optimization is difficult due to narrow therapeutic indices and inter-individual variability in metabolism. In the absence of targeted drug delivery systems, therapeutic concentrations needed for antitumor activity can be very near to the toxic threshold in healthy tissues. In order to achieve maximum benefit with minimum harm, careful pharmacokinetic modelling, therapeutic drug monitoring, and formulation-guided dose titration are necessary when integrating essential oils into oncology protocols (32, 213).

### 5.4 Active targeting strategies

Both cancer cells and tumor vasculature overexpress specific receptors, which can be exploited to successfully create functionalized surfaces on nanocarriers through covalent bonding of ligands such as folate, transferrin, hyaluronic acid and RGD peptides (248). This approach leads to increased intracellular delivery via receptor-mediated endocytosis, while avoiding non-specific pathways for uptake. In addition to ligand conjugation, additional strategies to actively target cancer include the use of antibody fragments (e.g. scFv), aptamers and dual-targeting mechanisms (249). To minimize off-target effects, stimuli-responsive linkers that cleave exclusively in the acidic/reductive tumor environment allow for ligand activation to be restricted to the disease site. When combined, these surface modifications enhance the therapeutic index, reduce drug exposure to healthy tissues and provide specific dosing for nanocarriers with actively targeted compounds, such as EO. Nevertheless, the translation of these types of systems to clinical practice will also need to overcome emerging resistance mechanisms such as the mutations associated with hepatocarcinogenesis (rtA181T mutation) (250), EGFR Exon 20 insertions (251) and EGFR C797S mutations (252), and therefore require further investigation. To overcome these mutations, combining EO with additional therapeutics or next-generation nanocarrier systems will likely be required to reverse resistance and maintain effective therapies.

### 5.5 Manufacturing and regulatory challenges in clinical translation

Beyond the inherent pharmacokinetic limitations of EOs, the translation of nanoformulated EO therapeutics into clinical practice faces substantial manufacturing, reproducibility, sterilization, and regulatory challenges. Large-scale manufacturing and translation of EO nanocarriers are challenged by maintaining batch-to-batch reproducibility in particle/droplet size, polydispersity, stability, and overall formulation performance due to process- and scale-dependent variability (18, 238, 253). The inherent variability in EO composition due to factors such as plant cultivar, geographical origin, harvesting season, and extraction method introduces additional complexity (254). Standardization of chemotypic profiles using validated analytical techniques (GC-MS, HPLC, NMR) is essential but challenging given the diverse and complex nature of EOs (255).

Sterilization represents another critical hurdle for clinical translation. Conventional terminal sterilization (autoclaving or ethylene oxide) is often unsuitable for EO nanoformulations due to thermal/oxidative degradation of volatiles and nanocarrier alterations (241, 256). Alternative approaches, including aseptic processing, filtration through 0.22 µm membranes, and gamma irradiation, require careful optimization to maintain product integrity and sterility assurance. Regulatory frameworks for EO-based nanomedicines remain ill-defined and inconsistent across jurisdictions. The classification of botanical EOs varies greatly and often falls outside oncology efficacy requirements, being categorized instead as dietary supplements or traditional medicines (257). The current regulatory framework requires modification to accommodate complex botanicals through tiered approval pathways, biomarker-driven endpoints, and integration of real-world evidence. Harmonization of guidelines from EMEA, FDA, and WHO is essential to improve global clinical acceptance and attract pharmaceutical investment (257). Additionally, the demonstration of safety and efficacy for each new chemotype or nanoformulation combination necessitates substantial investment in preclinical toxicology, pharmacokinetic profiling, and clinical trials that may be cost-prohibitive for natural product developers.

## 6 Advanced delivery systems for essential oils in cancer therapy

Advanced nanodelivery methods play an integral role for the delivery of EOs in cancer therapeutics (18), improving pharmacokinetics, and increasing targeting capabilities. Nanocarriers can provide structural protection against oxidation, improve solubility (7) by dispersing EO in aqueous solutions, and allow controlled release of EOs from the vehicle. The enhanced permeability and retention effect can be exploited to allow passive accumulation of nanocarriers at the site of the tumor, while functionalizing the surface of the nanocarrier allows active targeting to the tumor cells using the overexpressed surface receptors. Nanocarriers not only improve the index of elasticity of the EO (258), but also allows for co-encapsulation of EO bioactives and chemotherapeutic or immunomodulator agents to facilitate combination therapy. Each type of nanocarriers such as lipid-based, polymeric, emulsion, and inorganic has unique benefits pertaining to stability, loading capacity, and biocompatibility (259). The specific parameters such as size, surface charge, and degradation profile during the synthesis of nanocarrier can be tailored to achieve the circulation time and penetration into the tumor site after injection. As the field of formulation science continues to evolve, the use of nanocarriers to deliver EOs is being transitioned from proof-of-concept to clinically relevant precision therapeutic products, serving as a bridge between traditional phytochemistry and modern oncology.

### 6.1 Lipid-based nanocarriers

The most advanced systems currently used in delivering EOs are lipid-based nanocarriers (260). Such systems use biocompatible phospholipids to encapsulate lipophilic terpenoid compounds. Lipid-based nanocarrier systems have an inherent structural similarity to biological membranes, allowing for enhanced cellular uptake. The lipid-based nanocarrier systems can passively or actively targeted tumors, they can protect volatile compounds from degradation (260, 261) and can allow for their sustained release. The continuous optimization of both the lipid composition and surface modification of the lipid-based nanocarriers has greatly improved the predictability of the pharmacokinetics of the systems, making lipid-based nanocarriers the foundational delivery systems for precision phytotherapies.

#### 6.1.1 Liposomes and phytosomes

With a hydrophobic core/interlamellar spaces, a liposome (262) can hold EO constituents efficiently. The liposomes have great biocompatibility, tunable surface charges, and a high degree of PEGylation (263) that all cause them to have less turnover time (that reduces opsonization in the systemic circulation than traditional methods of delivery. Phytosomes are a special subclass of liposomes, consisting of molecular complex formed between the phospholipids in a liposome and a polyphenolic EO constituent (264). Phytosomes have for enhanced membrane permeability and intracellular delivery via endocytosis. Studies carried out *in vitro* have shown that liposomal limonene shows enhanced uptake and thus cytotoxicity in glioma models (e.g., against U87MG cells) (265). Moreover, phytosomal eugenol also showed improved s delivery in non-tumor contexts (e.g., wounds) (266). Further studies have also demonstrated that liposomal limonene and phytosomal eugenol show improved delivery and/stability in tumour models (43) whereas the free compounds undergo rapid hepatic metabolism (267). Nonetheless, the stability of liposomes is sensitive to pH changes and temperature fluctuations. The development of cationic liposomes has allowed for enhanced endosomal escape (268) and thus improved cytosolic delivery of EO bioactives. The co-encapsulation of bioactive compounds alone (269) or chemotherapeutics with EO bioactives within liposomes lead to synergistically enhanced tumor suppression, particularly with resistant metastatic melanoma (270). This synergistic potential is mechanistically supported by the identification of Integrated Stress Response (ISR)-associated prognostic genes in melanoma, such as GPX2 and DTL, which are linked to oxidative stress adaptation, metabolic reprogramming, and therapeutic resistance (271). Through modulating the ISR pathway—a core adaptive mechanism that promotes tumor survival under stress—EO nanoformulations may counteract the very stress-adaptive pathways that drive melanoma progression and immunotherapy failure, providing a molecular rationale for their enhanced efficacy in resistant settings. The use of phytosomes also improves the pharmacokinetic consistency (266) of EOs by preventing crystallization and improving solubility. Manufacturing of liposomes moves into GMP-compliant phase I/II clinical trials (272) and gradually EO delivery systems is also becoming more clinically viable. The clinical relevance of such liposomal co-delivery strategies is further underscored by the approval of the liposomal immunomodulator mifamuride (Mepact) for osteosarcoma, demonstrating that lipid-based nanocarriers can achieve regulatory success in oncology and providing a translational framework for EO-based formulations (273).

### 6.2 Polymeric nanoparticles and micelles

Amphiphilic block copolymers such as PLGA and PEG-PLA are employed in the synthesis of polymeric nanoparticles and micelles that contain both a hydrophilic and a hydrophobic components (274) to solubilise lipophilic constituents of EOs. The hydrophobic portion (the core) of the particle can be used to load terpenoids, whereas the hydrophilic portion (the corona) could provide steric stabilization and slow the circulation time within the body (control release). These systems can be designed for tunable degradation times such that they may provide sustained or stimuli-responsive release of drug in an acidic tumor microenvironment. PLGA-based polymeric nanoparticles have demonstrated high drug loading (over 80% encapsulation efficiency) of limonene (275) and eugenol (276) and the *in vitro* release profiles could be modified by changing the molecular weight of the polymer and the ratio of lactide:glycolide in the copolymer. Likewise, another similar nanodelivery systems micelles can self-assemble upon reaching the critical micelle concentration and form nanoscale (< 50 nm) structures (277). Functionalizing the surface of these delivery system with targeting ligands such as transferrin and hyaluronic acid, specific uptake through receptor-mediated endocytosis can be achieved in tumors that are positive for CD44^+^ or TfR^+^ (278). The above mentioned studies have shown that there is reduced systemic toxicity and improved tumor-to-liver ratio *in vivo* as compared to free oils and that polymeric systems allow for the co-encapsulation of EO bioactives along with chemotherapeutics (18). However, the degradation products resulting from polymers can provoke mild inflammatory responses (279), and therefore they must be optimized for biocompatibility prior to utilizing them as therapeutics. Recent developments in stimuli-responsive polymers, including pH-sensitive, redox-sensitive, and enzyme-cleavable systems, provide for the precise release of the therapeutic payload after endosomal acidification or glutathione exposure within the intracellular environment. With an increasing number of polymeric nanocarriers receiving regulatory approval for use in oncology therapies, EO micelles and nanoparticles could provide multiple options for precision delivery of therapeutics through multi-targeted approaches.

### 6.3 Nanoemulsions and self-nanoemulsifying drug delivery systems

Nanoemulsions and self-nanoemulsifying drug delivery systems (SNEDDS) are thermodynamically or kinetically stable colloidal systems (280) that could provide significant solubility and bioavailability enhancements for EOs. The composition of these systems is oil, surfactant, and co-surfactant, and they spontaneously create submicron droplets (<200 nm) when diluted with water. This mimics normal physiological lipid digestion. The oil phase can be used to contain the majority of the EO, while the surfactants could help to stabilize the interfacial tension between the oil and water and prevent coalescence. *In vitro* dissolution studies have demonstrated that EOs are released from SNEDDS quickly and completely (281, 282), thus, there are no barriers to absorption due to solubility limitations. The lymphatic absorption of SNEDDS formulations is superior to that of conventional formulations (283), providing a distinct advantage over conventional formulations in that they are able to bypass first-pass hepatic metabolism and provide greater systemic exposure. *In vivo* pharmacokinetic profiles demonstrate a significantly larger area under the curve (AUC) (2–7-fold) and a longer T_max_ time to reach peak concentration when utilizing terpenoid components (284). The addition of cationic or anionic surfactants S-SNEDDS (self-nano-emulsifying drug delivery systems)modulate surface charge improves mucosal adhesion and targeting of tumor cells (285). Co-encapsulation of chemotherapeutics with terpenoids (e.g., eugenol) enhances cytotoxicity in tumor models (285), thereby enhancing the efficacy of such combinations. High concentrations of surfactants have been shown to potentially cause hemolysis or gastrointestinal irritation, therefore requiring optimization of generally recognized as safe (GRAS) excipients prior to use. Other recent advancements in solid SNEDDS using silica or mannitol as matrix materials have published data that will improve stability for shelf life and the development of tablet/capsule dosage forms (286). As the standardization of manufacturing protocols occurs, the use of nanoemulsion and S-SNEDDS platforms would continue to provide scalable and orally available delivery systems for the use of precision EO-based phytotherapy.

### 6.4 Inorganic nanocarriers

A variety of inorganic nanocarrier systems (e.g., mesoporous silica, gold, iron oxide nanoparticles) have unique characteristics that make them attractive for EO delivery (287). They display high surface area, tunable porosity, and stimuli-responsive properties (288) are suitable for EO delivery The specific functionalization of nanoparticles’ surface allows for precise targeting and imaging applications (287, 288). The kinetic profiles exhibited by these nanoparticles have enabled a high degree of control over sustained release, as well as multimodal theranostic applications (289) (e.g., combining EO delivery with photothermal or magnetic targeting). Biocompatibility and clearance pathways for inorganic nanoparticles are still actively being explored for improvement; however, recent biodegradable polymer coatings have led to improved safety profiles for these systems.

#### 6.4.1 Mesoporous silica nanoparticles

MSNs have very large pore volume and surface area which provide high-capacity loading for EOs, thus are advantageous over other types of carriers for EO delivery (290). They have pore sizes of 2–50 nm and surface area >900 m²/g (291). Pore sizes and surface chemistries can be designed and manufactured by sol-gel synthesis to selectively encapsulate monoterpenes or sesquiterpenes. The silica-based framework provides protection against oxidation and premature degradation of volatile compounds (292). This occurs while allowing for gated release of EOs through pH-sensitive gating mechanisms such as surface-capped polymers or disulfide linkages triggered by tumor microenvironment. *In vitro* studies demonstrated that the sustained diffusion of EOs occurs for 72 hours (293), with accelerated release of EOs occurring when exposed to solutions of pH 5.5-6.5 (294), or in presence of high concentrations of glutathione, mimicking intracellular compartments. Functionalization of MSNs with folic acid or RGD (arginyl-glycyl-aspartic acid) peptides promotes receptor-mediated endocytosis in FRα^+^ or integrin-overexpressing solid tumors (295). Therefore, it is possible to significantly enhance the intracellular accumulation of EO cargo, which potentiates its pro-apoptotic and anti-proliferative effects while minimizing premature clearance and non-specific uptake in healthy tissues. Biocompatibility of the MSNs can be optimized through PEGylation or chitosan coating to reduce opsonization and increase circulation half-lives (296). Studies indicate that MSNs enhance tumor accumulation and reduce hepatic clearance (297) however its comparison with free EOs is hardly investigated. MSNs can also be used for co-delivery of imaging agents (298) or radiosensitizers with EOs, which has important implications for theranostic applications. New advances in the development of biodegradable organosilica frameworks will allow for the complete renal clearance of MSNs and minimize long-term toxicity (299). As the synthesis of MSNs becomes standardized as per good manufacturing practices, MSN-based EO delivery platforms could also be custom-designed, precision-engineered carriers suitable for oncology applications.

#### 6.4.2 Metal and metal oxide nanoparticles

Nanoparticles of metal and metal oxide (e.g. gold, silver, iron oxide, or zinc oxide) can serve as a multi-functional builder for the delivery of EOs, allowing transport of therapeutic payloads while also providing imaging, photothermal or catalytic properties. One example includes the use of gold nanoparticles (AuNPs) for photothermal therapy guided by surface plasmon resonance (300). These can be used for the localized release of EOs as well as ablation of the tumor. Superparamagnetic iron oxide nanoparticles allow for magnetic targeting and contrast for MRI imaging to improve spatial targeting (301). Silver and zinc oxide NPs have intrinsic antimicrobial and pro-oxidative properties that may work synergistically with the bioactive component of EOs (302). There is much still to be done regarding the long-term accumulation and clearance of all the types noted; however, due to the advances made in biodegradable coatings and control of size during synthesis, toxicity is much lower than previously measured. The applications of these various platforms are precision theranostics, thereby integrating the use of EO phytotherapy with real time imaging and stimulus-responsive delivery.

## 7 Synergistic combinations and multidrug resistance reversal

EO can enhance the efficacy of standard anti-cancer chemotherapeutics and reduce the toxicity associated with these drug therapies while mitigating the development of chemoresistant tumors as well (303). Because EOs possess multiple targeting mechanisms compared to single-agent chemotherapeutics, they can reactivate apoptosis and modulate the tumor microenvironment (8). It has been observed that EO acts by inhibiting the activity of efflux pumps (e.g., P-glycoprotein/ABCB1), DNA repair pathways, and survival signalling pathways (e.g., PI3K/Akt, NF-κB, MAPK) within a chemo-resistant tumor (18, 304). Thereby reversing the development of multidrug-resistant tumors and enhance the treatment effect of immunotherapy and/or radiotherapy. Therefore, implementing rational combinations of EO to exploit their respective synergistic effects could be critical in advancing precision phytotherapy for use in clinical oncology.

### 7.1 Essential oils combined with conventional chemotherapeutics

EOs increase the effectiveness of traditional chemotherapy as a result of both pharmacodynamic and pharmacokinetic interactions (8). Terpenoid-rich fractions enhance the sensitivity of cancer cells to DNA-damaging agents by inducing apoptosis and modulating DNA damage response pathways (305, 306). Thereby reducing levels of glutathione, which is important for detoxifying platinum- and alkylating-based chemotherapeutics. Both limonene and eugenol can inhibit the expression of P-glycoprotein and related efflux pumps (307, 308), thereby increasing the intracellular accumulation of doxorubicin, paclitaxel, and vincristine in cancer cells. Synergistic cytotoxicity has been demonstrated *in vitro* in breast, ovarian, and colorectal cancer cells (CI < 1) with dose reduction of chemotherapeutic agents (8, 32).

Quantitative analyses of the synergistic interactions between EO constituents and FDA-approved chemotherapeutic agents have been rigorously evaluated using Combination Index (CI) calculations based on the Chou-Talalay method. These analyses consistently demonstrate synergistic cytotoxicity (CI < 1) across multiple cancer types. d-Limonene exhibits anticancer activity against prostate cancer cells and has been reported to enhance the effects of chemotherapeutic agents like docetaxel (45, 309). The combination of rosemary essential oil nanoemulsion (REO/NE) with mitomycin C (MC) demonstrated synergistic activity on MCF-7 breast cancer cells, with a CI of 0.79 at the IC50, and a stronger synergy (CI = 0.60) at higher concentrations (310). Furthermore, β-caryophyllene although lacking cytotoxicity as a standalone agent, was shown to enhance the cytotoxic activity of paclitaxel across various cancer cell lines by increasing cell membrane permeability (311). This effect was particularly notable in DLD-1 colorectal cancer cells, where β-caryophyllene augmented paclitaxel activity by approximately tenfold, attributed to enhanced intracellular drug delivery (309). The combination of *Lippia alba* EO (enriched in citral) with clofarabine also exhibited remarkable synergistic anti-leukemic performance while preserving the integrity of healthy cells (312).

Synergistic effects have also been documented for citral in combination with doxorubicin, where CI values <1 confirmed enhanced apoptotic induction in lymphoma (32). Mechanistically, these synergistic interactions are attributed to the ability of EO constituents to downregulate NF-κB and PI3K/AKT survival signaling pathways (313), thereby eliminating compensatory pathways associated with chemotherapy-induced cellular stress. The downregulation of P-glycoprotein and related efflux pumps by EO constituents further increases intracellular accumulation of chemotherapeutic agents such as doxorubicin, paclitaxel, and vincristine (307, 308). Importantly, *in vivo* studies combining EOs with platinum-based chemotherapeutics have demonstrated reduced cardiotoxicity and nephrotoxicity alongside improved antitumor efficacy (314). The use of nanoencapsulation for co-delivery of EOs and chemotherapeutics in single carriers further enhances synergistic effects through enhanced tumor accumulation and controlled release profiles (18).

Mechanistically, the constituents of EOs downregulate the NF-κB and PI3K/AKT survival signaling pathways (313), eliminating compensatory pathways associated with chemotherapy-induced cellular stress. Proteomic analysis reveals increased activation of caspase-3, -8 (233) and decreased expression of cyclin D1 (213) in cells treated with EO components suggesting their positive impact when administered in combination with chemotherapeutic agents. *In vivo* studies have shown improved antitumor efficacy and survival and a reduction in cardiotoxicity and nephrotoxicity when treated with essential oils and chemotherapeutic agents (314). The use of nanoencapsulation for the delivery of both EOs and chemotherapeutics in a single carrier could further enhance their synergistic effects through enhanced accumulation in the tumor. Liposomal doxorubicin in combination with β-caryophyllene provide superior cardiac safety and enhance the apoptotic response in drug-resistant cancer cells (315, 316). Pharmacokinetic studies provide evidence that EOs could influence the metabolism of chemotherapeutic agents possibly via the CYP450 enzyme system (25). In another study by Martile et al. (317), combination of EO from *Melaleuca alternifolia* with targeted cancer therapies such as dabrafenib and/or trametinib presents promising biological effects preventing tumor genesis or progression among diverse tumor histotypes. Emerging studies suggests that probiotics could also contribute towards management of cancer and associated complexities (318). Combination of EO with probiotics could shed further light in its applicability in cancer therapy. Further, careful therapeutic drug monitoring will be required to ensure the safe translation of these therapies into the clinical setting to avoid adverse drug interactions. EO is a rational avenue for using combinations of EO-chemotherapies to mitigate dose-limiting toxicities and restore sensitivity to drugs, and enhance therapeutic indexes; this positions precision-based phytotherapy as a potential adjunct to many of today’s modern oncology regimens.

### 7.2 Combination with immunotherapy and radiotherapy

EO shows radiosensitizing effects and immune modulation in cancer model which is well described by Samaila et al. (319), and Sandner et al. (320), in their research. The components of EO could modulates both MHC-I/II expression and co-stimulatory molecule (CD80/86) expression on dendritic cells (321) leading to better T-cell priming and enhanced infiltration of cytotoxic lymphocytes. A component of EO such as eugenol could influence PD-L1 expression *in vitro* through inhibition of the STAT3 pathway (142), contributing towards reversing T-cell exhaustion and enhancing efficacy of the anti-PD-1/PD-L1 *in vivo*. Other *in vivo* studies demonstrated an increase in CD8^+^ TILs and a decrease in Tregs following EO-based immunotherapy. The synergy between EO and radiotherapy could be mediated by the ability of EO to both amplify ROS and inhibit DNA repair. Furthermore, sesquiterpene lactones (e.g., alantolactone, costunolide) induce apoptosis via glutathione depletion, ROS generation, and mitochondrial dysfunction in cancer cells (322). Thereby it is possible that these compounds could inhibit double-strand break repair and enhancing the induction of apoptosis by radiation when administered in combination with radiotherapy. Overall, EO-immuno/radio combinations represent a multi-faceted approach to overcoming treatment resistance and improving long-term tumor control.

### 7.3 Overcoming ABC transporter–mediated multidrug resistance

Chemotherapy resistance results from the efflux of chemotherapeutic agents from tumor cells by ABC transporters [P-glycoprotein (P-gp/ABCB1), BCRP (ABCG2), and MRP1 (ABCC1)] (323). The constituents of EOs inhibit these transporters by direct binding or functional modulation (324). Terpenoids such as citral is capable of inhibiting P-gp ATPase activity (325), thus increasing the number of chemotherapeutic drugs retained within the cells. The use of EOs in conjunction with nanoencapsulated formulations helps to maintain inhibition of ABC transporters by limiting the compensatory upregulation of these transporters. Additionally, when combined with transporter inhibitors, the use of EOs could possibly enhance the effectiveness of these agents and create a novel approach to restore chemosensitivity in patients with resistant tumors.

## 8 Preclinical and clinical evidence

There are many studies that support the anticancer properties of EOs through extensive preclinical studies as well as emerging data from laboratory studies (238). The majority of preclinical studies have indicated that EOs are multi-targeted agents, have the ability to induce apoptosis in many different types of cancer, and can inhibit metastasis in laboratory models of cancer (32). Although there are limited numbers of clinical studies with EOs, early studies and safety profiles demonstrate good tolerability and appropriate pharmacokinetics for most EOs. Bridging preclinical and clinical studies is a key factor in establishing the use of essential oils as an adjunctive therapy in the treatment of cancer.

EO have been shown to have dose dependent cytotoxic effects in laboratory studies across a variety of cancer cell lines; IC50 values range from 10-200 µg/mL depending on the profile of the constituents of the EO and the sub-type of tumor (31, 32). Mechanistic studies confirm that EOs induce apoptosis through activation of caspases, induce arrest at the G2/M phase of the cell cycle (326) and possibly suppress migration/invasion of cells. Three-dimensional spheroid and organoid models are better able to reflect the complexity of the tumor microenvironment and show improved penetration of EOs and sustained cytotoxicity as compared to two-dimensional cultures. *In vivo* studies in xenograft and syngeneic models show a significant inhibition (40-70%) of tumor growth in the presence of systemically and locally administered essential oils (207, 238, 327). Nanocarrier delivery systems improve the pharmacokinetics resulting in not only ~2.8–2.9 fold higher cellular uptake of EO in tumors compared to native EO along with longer half-lives as well (328). Histopathological studies show decreased microvessel density, increased indices of apoptosis and normalized immune infiltration in tumors treated with EOs (329, 330). Studies examining metastasis show a significant decrease in colonization of the lung and liver when EOs are given prophylactically (331, 332). Studies examining toxicity of EOs indicate that there are very few off-target effects at clinically relevant dosages with the greatest concern being from hepatic metabolism at very high concentrations (333). Combinations of EOs with chemotherapy and immunotherapy produce synergistic regression of tumors and prolong survival (8). Standardization of extraction, dosing and formulation of EOs remains critical to produce reproducible results. As current innovations move forward through preclinical testing with more humanized models and human derived xenografts the efficacy of EOs data could be used more frequently to develop clinical trial designs and dosing strategies.

### 8.1 Clinical trials and safety profiles

The current status of clinical assessments of EOs for cancer patients has mostly involved symptom management or improving patient’s quality of life. Evaluating their pharmacokinetic profiles/safety instead of determining whether there is an independent effect on tumor growth when used as a monotherapy needs further investigations. Phase I clinical trials using oral limonene (334, 335) and topical eugenol formulations (336) are generally reported to have a safe and well-tolerated profile with mild side effects. These mild side effects being largely limited to gastrointestinal upset or localized contact dermatitis, particularly at higher doses. The pharmacokinetic assessments suggest that these EOs are rapidly absorbed and undergo a significant first-pass effect through the liver prior to circulating in the bloodstream, thus having a short half-life (247). This indicates that development of a suitable delivery system to provide patients with better sustained therapeutic levels of the EOs may be warranted. In initial combinations of EO formulations with standard chemotherapeutic agents, some benefits of the EOs on several patient-reported outcomes have been noted, including decreased nausea and maintenance of immune function (337). However, mainly due to the small number of individuals participating in these studies, the variation in composition of different EOs, and the lack of quality control in product formulations, efficacy endpoints for the EOs have largely remained exploratory. In recent small pilot studies that have attempted to use nanoparticulate formulations of EOs (Listed in **Table 1**), there have been preliminary reports documented on their improved pharmacokinetics in terms of dose proportionality and absorption, thus providing evidence that further development and improvement of EO formulations may be warranted. Nonetheless, continued need for well-controlled, large-scale clinical trials that will confirm the anticancer effects of formulations including EOs remains to be conducted. To improve reproducibility in trials and to gain acceptance from regulatory agencies, a consistent chemotype of EOs, accurate and precise dosages, and robust quality controls using validated analytical techniques must be implemented. As the clinical trial infrastructure evolves to meet the unique needs of precision phytotherapy, the role of EOs in clinical trials is anticipated to shift from primarily offering support through the clinical trial infrastructure, to offering a robust source of evidence-based, multi-target and mechanistic approaches guided by extensive preclinical research and established data demonstrating both safety and efficacy.

### 8.2 Clinical status of EO Nanoformulations

Despite the extensive preclinical evidence supporting the anticancer potential of EO nanoformulations, their clinical translation remains in its infancy (**Table 2**). A critical challenge is the very low number of clinical trials evaluating nanoformulated EO-based therapeutics (3, 12). Current clinical assessments of EOs in cancer patients have predominantly focused on symptom management or quality-of-life improvement rather than evaluating independent antitumor efficacy as monotherapy. Phase I clinical trials using oral limonene formulations (334, 335) and topical eugenol formulations (336) have generally reported safe and well-tolerated profiles with mild side effects, primarily limited to gastrointestinal upset or localized contact dermatitis at higher doses.

**Table 2:** Comprehensive summary of major essential oil constituents, botanical sources, molecular targets, cancer types, experimental models, and level of scientific evidence.

Constituent	Class	Botanical sources	Primary molecular targets	Cancer types studied	Experimental models	Level of evidence
Limonene	Monoterpene	*Citrus peel oils* (Citrus spp.)	PI3K/AKT/mTOR, NF-κB, RAS prenylation, p21	Breast, liver, colorectal, glioma	*In vitro*: MCF-7, MDA-MB-231, Caco-2, U87MG; *In vivo*: xenograft models; Phase I clinical trials	Clinical (Phase I) (334, 335)
β-Caryophyllene	Sesquiterpene	Copaiba oil, *Origanum vulgare*, cloves	CB2 receptor, NF-κB, JAK/STAT3, VEGF	Breast, colon, pancreatic, melanoma	*In vitro*: *In vivo*: xenograft; Combination with paclitaxel (CI < 1)	Preclinical (*in vitro* + *in vivo*) (47, 48, 81, 145)
Thymol	Monoterpenoid	Thyme (*Thymus vulgaris*), oregano	NF-κB, STAT3, ROS modulation	Breast, gastric, lung	*In vitro*: MCF-7, AGS; *In vivo*: Murine models	Preclinical (*in vitro* + *in vivo*) (122, 234)
Carvacrol	Monoterpenoid	Oregano (*Origanum vulgare*), thyme	NF-κB, STAT3, PI3K/AKT, apoptosis	Breast, colon, lung	*In vitro*: MCF-7, HCT-116; *In vivo*: Xenograft models	Preclinical (*in vitro* + *in vivo*) (6, 234)
Citral	Monoterpenoid	Lemongrass (*Cymbopogon citratus*), lemon balm	Src/STAT3, apoptosis	Small-cell lung cancer (SCLC)	*In vitro*: LU134AM, LU135, LU165, MN1112 SCLC cell lines	Preclinical (*in vitro* + *in vivo*) (236)
Geraniol	Monoterpenoid	Palmarosa, rose, citronella, *Thymus daenensis*	CDK2/CDK4, cyclin D1/E/A, cell cycle arrest Cyclin D1, Cyclin E, Cyclin A; G0/G1 cell-cycle arrest; HMG-CoA reductase inhibition	Breast	*In vitro*: MCF-7 formulation	Preclinical (*in vitro*) (107)
Cinnamaldehyde	Phenylpropanoid	Cinnamon (*Cinnamomum verum*)	JNK, tubulin polymerization, spindle assembly checkpoint	Breast, colon	*In vitro*: MDA-MB-231, A549; *In vivo*: xenograft models; CS-CEO nanoparticles	Preclinical (+ *in vivo*) (105, 124)
α-Pinene	Monoterpene	*Pine*, *eucalyptus*, rosemary	TGF-β/Smad, ROS, mitochondrial dysfunctionPI3K/Akt/mTOR, oxidative stress	Breast, lung, liver	*In vivo*: Liver fibrosis model	Preclinical (*in vivo*) (150)

Note on Level of Evidence: “Clinical” indicates evidence from human trials; “Preclinical (*in vitro* + *in vivo*)” indicates studies in both cell culture and animal models; “Preclinical (*in vitro*)” indicates cell-based studies only.

Pharmacokinetic assessments from these trials indicate that EOs are rapidly absorbed and undergo significant first-pass hepatic metabolism, resulting in short half-lives (247). This pharmacokinetic profile underscores the rationale for developing nanoformulated delivery systems to achieve sustained therapeutic concentrations. Preliminary reports from recent small pilot studies utilizing nanoparticulate formulations of EOs have documented improved pharmacokinetics in terms of dose proportionality and absorption, suggesting that further formulation development is warranted (238, 327).

However, it must be acknowledged that nanoformulated EO therapeutics have not yet demonstrated clinical efficacy in Phase II or Phase III trials. The path to clinical translation faces significant hurdles, including variability in EO composition, lack of standardized quality control, and the complexity of regulatory classification for complex natural mixtures. The heterogeneity in nanoformulation designs, ranging from lipid-based nanoparticles to polymeric micelles and inorganic carriers, further complicates clinical development. While liposomal delivery systems for conventional chemotherapeutics have achieved clinical approval and are progressing to GMP-compliant manufacturing (272), EO-based nanomedicines remain predominantly at the proof-of-concept stage (18). The progression of EO nanoformulations into the clinical pipeline will require systematic pharmacokinetic/pharmacodynamic optimization, thorough toxicological evaluation, and well-controlled clinical trials using standardized formulations.

## 9 Standardization and quality control

Rigorous phytochemical profiling (GC/MS, HPLC, NMR) and quality control protocols (volatile degradation products, oxidative by-products and contaminant levels) are fundamental to the standardization of EOs (255). To ensure batch-to-batch consistency of EOs and reproducibility of therapeutic outcomes, standardized reference standards and validated analytical methods are necessary for regulatory compliance and clinical trial reliability. The classification of EOs by regulatory authorities varies greatly and often falls outside oncology efficacy requirements (e.g., dietary supplements, traditional medicine). Moreover, the classification of botanical EOs requires modification to current regulatory frameworks through the use of tiered approval pathways for complex botanicals, biomarker-driven endpoints, and integration of real-world evidence into the approval process. The harmonization of guidelines given by EMEA, FDA and WHO will improve the global clinical acceptance of EOs and attract pharmaceutical investment in EOs (257). Future use of EOs in phytotherapy will leverage the use of pharmacogenomics, artificial intelligence (AI) in formulation design, and patient-derived organoids to develop personalized dosing. The integration of liquid biopsies and immune profiling in the development of EOs will facilitate the implementation of biomarker-matched EO regimens in patients (238), enhancing the therapeutic index and preventing acquired resistance to EOs. Advancements in precision phytotherapy will require ongoing investment in translational research and modernization of regulations.

## 10 Translational challenges and future perspectives

The path from preclinical promise to clinical reality for EO-based nanomedicines is significantly hindered by the limitations of current prediction methods. As highlighted by recent advances in computational biology, a critical bottleneck is the inability of conventional models to effectively integrate multi-level interaction data, leading to suboptimal predictions and high attrition rates in drug development (338). Despite strong mechanistic insights and substantial preclinical evidence, the clinical advancement of EO-based nanomedicines for cancer treatment continues to face several interrelated challenges. A primary obstacle is the intrinsic chemical complexity and compositional variability of EOs. Their phytochemical composition is greatly influenced by factors such as plant cultivar, geographical origin, harvesting season, storage conditions, and extraction techniques (254). Consequently, comprehensive phytochemical characterization using techniques such as GC/MS, HPLC, and NMR, together with standardized quality control measures for every batch, is essential to ensure reproducibility of therapeutic efficacy and the reliability of clinical trial outcomes (255).

The limited number and scope of clinical trials represent another critical bottleneck. Current clinical assessments of EOs in cancer patients have primarily addressed symptom management or quality-of-life improvement rather than evaluating independent antitumor activity (238, 337). The lack of well-powered, randomized controlled trials with biomarker stratification and standardized dosing regimens severely limits the evidence base required for regulatory approval and clinical adoption. Furthermore, the absence of harmonized regulatory frameworks across jurisdictions creates significant barriers for multinational clinical development programs.

From a formulation perspective, achieving consistent nanoparticle manufacturing at scale remains challenging. The complexity of multi-component EO mixtures complicates the optimization of drug loading, encapsulation efficiency, and release kinetics (18, 238). Sterilization of thermolabile EO nanoformulations without compromising active components requires further development. Additionally, the long-term stability of nanoformulated EOs under clinical storage conditions and their compatibility with administration routes (oral, intravenous, topical) require systematic investigation. The immunogenicity, biocompatibility, and clearance profiles of various nanocarrier systems also demand thorough evaluation, particularly for inorganic carriers such as mesoporous silica and metal nanoparticles (296, 299).

The economic viability of EO-based nanomedicines presents additional challenges. The high costs associated with standardized cultivation, quality-controlled extraction, rigorous phytochemical characterization, and complex nanofabrication processes may not be offset by the relatively low market value of botanical products. This economic disincentive discourages pharmaceutical investment in EO nanomedicine development. Furthermore, intellectual property protection for complex natural product mixtures and nanoparticulate formulations presents legal and strategic complexities.

To overcome these translational barriers, a coordinated, multidisciplinary effort is required. This includes the development of standardized reference materials and analytical protocols for EOs, the establishment of Good Manufacturing Practice (GMP) guidelines specifically for EO-based pharmaceuticals, the design of adaptive clinical trial frameworks that accommodate the multi-targeted nature of EOs, and the creation of regulatory pathways for complex botanical-nanoparticle combinations. Additionally, investment in pharmacogenomics and biomarker-driven patient stratification will enable precision matching of EO chemotypes to tumor molecular subtypes, enhancing therapeutic index and accelerating clinical adoption (238).

The integration of artificial intelligence (AI) in formulation design, the use of patient-derived organoids for preclinical efficacy assessment, and the application of liquid biopsies for real-time monitoring of treatment response represent promising avenues for advancing EO-based precision phytotherapy into mainstream oncology practice. With sustained investment in translational research and regulatory modernization, EO-based nanomedicines have the potential to transition from laboratory promise to clinical reality as scientifically validated, multi-targeted adjunctive cancer therapies.

## 11 Conclusion

Overall, EOs, as a new class of precision-based phytotherapy, show promise in the treatment of cancer with documented anticancer effects in many different preclinical models. The bioactive components, terpenoids and phenylpropanoids, in EOs interact with overlapping oncogenic pathways that result in apoptosis induced cell cycle arrest, angiogenesis inhibition, metastasis and epithelial-mesenchymal transition suppression, and positive influence on the tumor microenvironment. Despite their anticancer potential, EOs have pharmacokinetic limitations due to their lack of chemical stability, systemic bioavailability, and tumor-specific accumulation. Because of the biodistribution properties and molecular weight of EOs, novel nanodelivery systems are being developed with the goal of achieving the optimal therapeutic window and enabling the means to combine EOs with standard-of-care chemotherapeutics or other biologics such as immunotherapy or radiotherapy. Various lipid-based, polymeric, emulsion-based, and inorganic nanomaterials with varying characteristics have been studied as potential carriers for EOs to achieve enhanced stability and bioavailability. Despite compelling mechanistic and robust data from preclinical testing, successful clinical translation of EOs to treat cancer is limited by multiple factors such as a lack of standardization of EOs; a lack of regulatory classification of EOs as complex natural mixtures; and a need for adequate power clinical trials that include biomarker stratification. To achieve successful integration of multi-component herbal products into the larger health care community, it is critical to address translational impediments through unified quality assurance criteria, advanced analytical validation, multidisciplinary co-operation between fields and disciplined use of adaptable regulatory mechanisms that address the needs of these products. The emerging paradigm of precision oncology with its focus on multimodality, low toxicity therapies is well-aligned with the integration of EO-based phytotherapy as scientifically valid, providing a plausible low-cost adjunctive treatment modality that can enhance patient quality of life. In order to determine the eventual appropriate position of EO therapy within contemporary personalised cancer treatment paradigms, ongoing financial support for scientifically rigorous clinical validation and ongoing product formulation innovation will be required.
